# Effect of conservative therapy for persistent postural-perceptual dizziness: a systematic review and meta-analysis

**DOI:** 10.3389/fpsyt.2025.1676218

**Published:** 2025-10-30

**Authors:** Yaqing Zheng, Ziyan Guo, Xinkun Liu, He Chen, Weijuan Gang, Huan Chen, Weiming Wang

**Affiliations:** ^1^ Department of Acupuncture, Guang’anmen Hospital, China Academy of Chinese Medical Sciences, Beijing, China; ^2^ Institute of Acupuncture and Moxibustion, China Academy of Chinese Medical Sciences, Beijing, China

**Keywords:** PPPD, persistent postural-perceptual dizziness, conservative interventions, systematic review, meta-analysis

## Abstract

**Objective:**

To evaluate the effect of conservative therapy in improving function and symptom for patients with Persistent Postural-Perceptual Dizziness (PPPD) in order to provide evidence for clinical practice.

**Methods:**

Randomized controlled trials (RCTs) assessing the effect of conservative therapy for PPPD were searched in 5 databases (CNKI, Wanfang, SinoMed, PubMed and EMBASE) up to 8^th^ March 2025. Risk of bias of included studies were assessed using Cochrane Risk-of-Bias (RoB) tool version 2. Meta-analysis was conducted where applicable.

**Results:**

Twenty-two studies (1,764 patients) were included in this review. For selective serotonin reuptake inhibitors (SSRIs) and vestibular rehabilitation therapy (VRT), the pooled estimates presented consistent results that the combined therapy had significant improvements on Dizziness Handicap Inventory (DHI) and Hamilton Anxiety Scale (HAMA) compared with single therapy. However, the certainty of the effect of cognitive behavioral therapy (CBT) and transcranial direct current stimulation (tDCS) were unclear due to limited number of studies and small sample size. The major concern of risk of bias of included studies laid to selection of reported results and randomization process. Certainty of all outcomes were judged to be moderate to very low by using the GRADE (Grading of Recommendations Assessment, Development and Evaluation).

**Conclusion:**

Conservative therapies, particularly SSRIs combined with VRT or CBT, could improve functional status and symptom severity in PPPD patients with favorable safety profiles. Based on current evidence, we recommend to prioritize SSRI plus structured VRT as treatment option for patients with PPPD.

**Systematic review registration:**

https://www.crd.york.ac.uk/prospero/, identifier CRD42024544565.

## 1 Introduction

First proposed by Staab and Ruckenstein in 2015, Persistent Postural-Perceptual Dizziness (PPPD) was later categorized in chronic vestibular syndromes according to the International Classification of Diseases (11th Revision) and characterized by persistent non-rotatory dizziness, postural instability, and hypersensitivity to motion or complex visual stimuli (1). PPPD affects a large number of patients, accounting for 10% of outpatient dizziness cases, especially among women aged 40–60 years. Apart from its unclear pathophysiology, diagnosis and treatment of PPPD is often challenging due to the significant presence of anxiety, depression, or autonomic dysfunction in PPPD patients, negative findings in laboratory or imaging examinations (2). Currently, the first line treatment for PPPD is selective serotonin reuptake inhibitors (SSRIs), including sertraline, escitalopram, and fluoxetine (3). However, the use of SSRIs is problematic due to its common side effects (nausea, vomiting, sleep disorders, and sexual dysfunction, etc.), slow-acting and possibility to exacerbate patient anxiety during the initial stages of treatment, as well as serotonin syndrome related to long-term use of SSRI (4). Other conservative therapies, including vestibular rehabilitation therapy (VRT), cognitive behavioral therapy (CBT), multimodal approaches (e.g. lifestyle modifications and patient education) and etc. can also improve PPPD symptoms to some extent (5). However, existing studies report inconsistent effect of above mentioned therapies and no systematic review conducted. Therefore, this study aims to synthesize evidence from RCTs to assess the effect and safety of conservative interventions for PPPD.

## 2 Methods

This systematic review was conducted in accordance with the Cochrane Handbook for Systematic Reviews of Interventions and reported following the Preferred Reporting Items for Systematic Reviews and Meta-Analyses extension statement for NMA (PRISMA-NMA) (**Supplementary File 1**). The study protocol was prospectively registered in PROSPERO (CRD42024544565).

### 2.1 Search strategy

Five electronic databases, including CNKI, Wanfang, SinoMed, PubMed, and EMBASE, were searched from inception to 8^th^ March 2025. Chinese keywords included 持续性姿势-知觉性头晕’ , ‘持续性姿势-感知性头晕’ , ‘慢性主观性头晕’ , ‘慢性前庭综合征’, ‘随机对照试验’, ‘随机’, ‘对照实验’ and ‘临床试验. English keywords included ‘PPPD’, ‘Persistent Postural-Perceptual Dizziness’, ‘Functional dizziness’, ‘Chronic subjective dizziness’, ‘randomized controlled trial’, ‘randomized’, ‘controlled trial’ and ‘clinical trial’. Both free words and subject headings were applied in search strategy for each database when necessary (**Supplementary File 2**).

### 2.2 Inclusion criteria

Studies were included if they met all of the following criteria: 1) Study design: Randomized controlled trials (RCTs) published in Chinese or English; 2) Population: Patients meeting diagnostic criteria of PPPD or chronic subjective dizziness (CSD); 3) Interventions: Conservative therapies, including but not limited to SSRIs, VRT, physiotherapy, acupuncture, etc.; and 4) Outcomes: any indicators assessing severity of PPPD symptoms, anxiety and quality of life, including Dizziness Handicap Inventory (DHI), Hamilton Anxiety Scale (HAMA), Hamilton Depression Scale (HAMD), Hospital Anxiety and Depression Scale (HADS) and etc.

### 2.3 Exclusion criteria

Studies were excluded if they met any of the following criteria: 1) duplicated publications of one study; 2) full-texts were not available or no analyzable outcome data; and 3) herbal medicine used alone or in combination with others in either groups, or comparison between different types of acupuncture, or different treatment frequencies or protocol of the same type of acupuncture.

### 2.4 Study selection and data extraction

Two researchers independently screened titles, abstracts, and full texts of all retrieved literature using EndNote X9. Discrepancies were settled by a senior reviewer. Data extracted included author information, characteristic of study population, sample size, study design, details of interventions, outcome measures, and adverse events.

### 2.5 Assessment of risk of bias and certainty of evidence

The Cochrane Risk of Bias Tool version 2 (RoB 2) was employed to assess methodological rigor of included studies through five domains: 1) randomization process; 2) deviations from intended interventions; 3) missing outcome data; 4) measurement of the outcome; and 5) selection of the reported result. Each study was categorized into three risk levels (low risk, high risk, or unclear risk) for every domain through standardized evaluation criteria. Two investigators independently conducted the risk of bias assessments followed by cross-checking, with discrepancies solved by a senior researcher.

GRADE (Grading of Recommendations Assessment, Development and Evaluation) approach was conducted to evaluate the certainty of evidence.

### 2.6 Statistical analysis

Included studies were categorized based on their types of interventions. For continuous variables, the mean difference (MD) was used to measure treatment effect with 95% CIs. For dichotomous variables, treatment effects were presented as a risk ratio (RR) with 95% CIs. Meta-analyses were undertaken to synthesize outcome data where appropriate. Whether a fixed or a random effect model was adopted was determined by the results of the χ^2^ test and I2 test for heterogeneity. An I^2^ value of 50% or more indicated a substantial level of heterogeneity. A p value of less than 0.05 (two-sided testing) was considered as statistical significance. RevMan software version 5.4.1 was used to conduct statistical analysis.

## 3 Results

### 3.1 Search results

**Figure 1:**
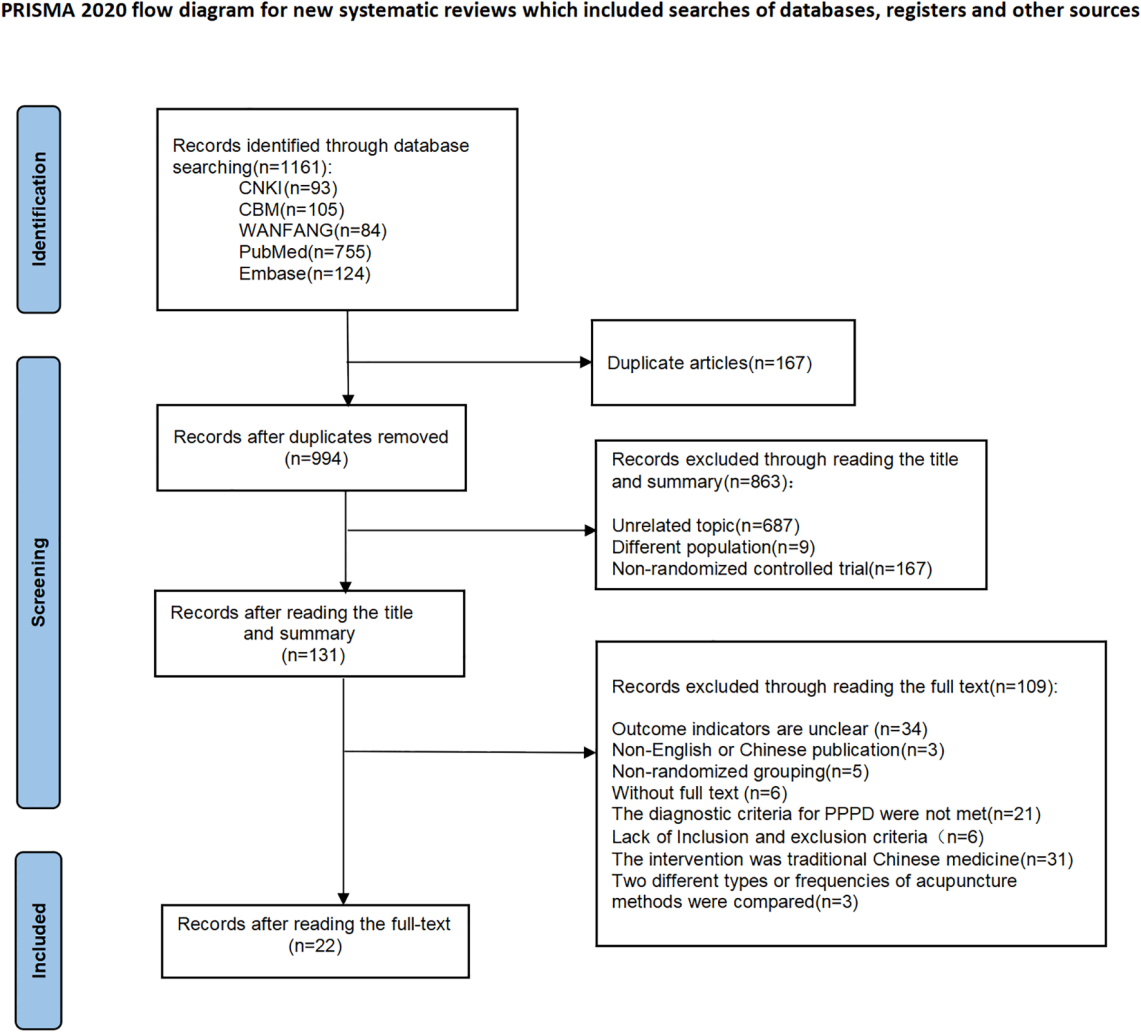
Study selection flow chart.

The initial database search yielded 1,161 records, comprising 256 papers in Chinese and 848 in English. After removing 167 duplicate records, 994 papers were kept and screened by title and abstract. Then 863 papers were excluded for not meeting the inclusion criteria, and 131 were left for full-text review. Finally, 22 studies of RCT were included for analysis. The study selection process is illustrated in **Figure 1**.

### 3.2 Study characteristics

There were 22 studies (n = 1,764 patients) included in this review. Among these, 12 studies with 1,052 patients (6–17) assessed the effect of SSRIs (including citalopram, sertraline, duloxetine, fluoxetine, and escitalopram), 3 studies with 185 patients (18–20) assessed the effect of non-SSRI medication, 5 studies with 463 patients (8, 21–24) investigated the effect of VRT, 1 study with 23 patients (25) examined the effect transcranial direct current stimulation (tDCS) and 1 study with 41 patients (26) evaluated the effect of CBT. All included trials employed parallel-group designs with comparable baseline data between groups (**Table 1**).

**Table 1:** Characteristics of included studies for PPPD.

Author Year Country	Diagnosis	Study design	Experimental group			Control group			period	Follow-up	Outcome measures	Adverse event
			Sample size (m/f)	Mean age	Intervention	Sample size (m/f)	Mean age	Intervention				
Hou 2021 China (21)	PPPD	RCT	43 (19/24)	53.3 ± 4.6	VRT+escitalopram, escitalopram initiated from 5mg daily and increased to 20 mg daily by 2 weeks, VRT same as control	43 (20/23)	52.8 ± 4.3	VRT, once daily, 20~25min/time	4-week	N/R	DHI, HAMA, HAMD,	N/R
Meng 2021 China (22)	PPPD	RCT	71 (31/40)	32.92 ± 5.59	SRT+NCC, SRT not specified; NCC same as the control.	71 (32/39)	32.85 ± 5.23	RRT+NCC, RRT not specified; NCC initiated from 1 capsule daily, increased to 1 capsule, twice daily when necessary.	1month	N/R	DHI	N/R
Zhang 2022 China (6)	PPPD	RCT	45 (20/25)	66.40 ± 5.45	sertraline+VRT, VRT 3 times daily, 20min/time; Sertraline same as the control.	45 (24/21)	66.50 ± 5.50	sertraline 25 mg/time, once daily from day 1 to 3, can be increased ≤ 100mg/day in 7-day when necessary.	6-week	N/R	SAS,SDS	N/R
Cao 2020 China (7)	PPPD	RCT	30 (14/16)	46.97 ± 12.72	duloxetine +biofeedback-CBT+VRT, duloxetine initiated from 30mg/day, and increased to 60mg/d when necessary. biofeedback-CBT+VRT same as the control.	30 (12/18)	47.27 ± 13.26	escitalopram+ biofeedback-CBT+VRT, escitalopram initiated from 5mg/day, increased to 10mg/day in 6-week; biofeedback-CBT twice weekly, 0.5h/session; VRT 3 times/day, 10min/session	6-week	N/R	DHI, HADS	N/R
Li 2021 China (8)	PPPD	RCT	30 (12/18)	55.21 ± 4.23	sertraline+ acupuncture, acupuncture & Lingnan fire needle at GV20, once/day; sertraline same as the control.	30 (13/17)	56.13 ± 3.25	sertraline, 50mg/time, once daily	4-week	N/R	DHI, HAMA, HAMD	N/R
Li 2022 China (27)	PPPD	RCT	40 (22/18)	53.37 ± 8.92	VRT+acupuncture(thumb-tack needle), acupuncture at ear acupoints (shenmen, heart, sympathetic, liver, kidney, subcortical, anterior pendulum),bilateral GV26, PC6,HT7, LR3, ST36, SP6, once/week; VRT same as the control.	40 (19/21)	55.37 ± 8.08	VRT 4 times/day	4-week	N/R	SSS, HAMA	N/R
Lin 2020 China (9)	PPPD	RCT	30 (9/21)	49.59 ± 15.13	fluoxetine+TES, TES 30min/time, once daily, 10-day for 1 course, 3-course in total; fluoxetine same as the control.	30 (10/20)	48.14 ± 16.42	fluoxetine 20mg/time, once daily	30-day	N/R	DHI HADS	N/R
Mo 2019 China (10)	PPPD	RCT	70 (34/36)	40.15 ± 2.61	escitalopram 1–2 tablets/day for 30 consecutive days.	70 (25/35)	40.22 ± 2.56	VRT not specified	30-day	N/R	DHI, HAMA HAMD	N/R
Yuan 2020 China (18)	PPPD	RCT	33 (16/17)	41.23 ± 5.63	deanxit+AGT, AGT: 100 mg/time, 3 times/day; deanxit same as the control	30 (12/18)	37.72 ± 7.15	deanxit 10.5mg/day	2-week	N/R	DHI	N/R
Zhao 2020 China (11)	PPPD	RCT	29 (–)	N/R	sertraline+VDT, VDT not specified, 20min/day; sertraline same as the control	27	N/R	sertraline, 25mg/day	6-month	6-month	DHI HADS	4 cases
Zhao 2021 China (12)	PPPD	RCT	29 (8/21)	51.41 ± 2.26	Citalopram+ acupuncture, acupuncture at EX-HN3, GV23, EX-HN5, GV20, EX-HN1, GB20, GB12, BL10, EX-B2 C3-5, GB39, HT7, SP6, LI4, LR3, 30mins/time, 3 times/week; escitalopram same as the control.	29 (5/24)	49.05 ± 4.74	escitalopram 1 tablet/time, once daily	8-week	N/R	DHI HAMA HAMD	N/R
Chen 2018 China (13)	PPPD	RCT	75 (38/37)	47 ± 11	Citalopram+ VRT+psychotherapy, VRT not specified, twice/day, 10-15mins/time; psychotherapy twice/week, 20mins/time; citalopram same as the control.	75 (39/36)	47 ± 10	citalopram 20mg/day	2-month	N/R	DHI HAMA HAMD	N/R
Zhou 2019 China (14)	PPPD	RCT	32 (13/19)	46.88 ± 9.36	citalopram+ biofeedback-CBT biofeedback 3 times/week, 30mins/time. CBT twice/week, 40mins/time. citalopram same as the control.	32 (15/17)	47.95 ± 10.84	citalopram, 1^st^ week 5mg/day, 2^nd^ week increase to ≤ 20mg once daily.	8-week	N/R	DHI	N/R
Xu 2023 China (19)	PPPD	RCT	30 (22/8)	56 (51.2-66.5)	betahistine+VRT, VRT not specified; betahistine same as the control.	30 (19/11)	58 (47-65)	betahistine 6mg/time, 3 times/day	8-week	N/R	DHI VSI	N/R
Edelman2012 Australia (26)	PPPD	RCT	20	N/R	CBT Once/week	21	N/R	wait-list control	3-week	6-month	DHI DSI SBI	N/R
Im 2020 Korea (25)	PPPD	RCT	12 (4/8)	47.8 ± 13.0	tDCS 20 mins/time, 15 times for 3 weeks	11 (4/7)	51.7 ± 13.1	sham tDCS 20mins/time, 15 times for 3 weeks	3-week	3-month	DHI ABC HDRS HARS	N/R
Teh 2024 Malaysia (23)	PPPD	RCT	30 (8/22)	44.77 (10.04)	home-based VRT, at least 10 mins/day	29 (10/19)	48.41 (7.33)	hospital-based VRT, at least 3 times/day	12-week	N/R	DHI EQ5D	N/R
Qi 2018 China (15)	PPPD	RCT	60 (28/32)	46.4 ± 6.3	Citalopram+ VRT+CBT, VRT 10mins/time, 3 times/day; CBT 40mins/time, 2 times/week; citalopram same as the control.	60 (27/33)	46.9 ± 7.1	citalopram 10mg, once daily	60-day	N/R	Anxiety score	N/R
Yang 2018 China (24)	PPPD	RCT	48 (20/28)	41.68 ± 5.79	VRT+citalopram +CBT, citalopram once daily, 10mg/day in the 1^st^ week, ≤20mg/day in the 2^nd^ week; CBT once/week; VRT same as the control.	48 (22/26)	40.75 ± 8.23	VRT not specified once daily	8-week	N/R	DHI HAMA HAMD	N/R
Cao 2024 China (16)	PPPD	RCT	37 (14/23)	56.47 ± 10.28	duloxetine+VRT, duloxetine 30 mg/day in the 1^st^ week, 60 mg/day from the 2^nd^ week; VRT not specified	37 (12/25)	56.19 ± 10.46	duloxetine same as experimental group	8-week	N/R	DHI HADS PSQI BBS MoCA	N/R
Liu 2024 China (17)	PPPD	RCT	60 (25/35)	57.30 ± 4.32	duloxetine+VRT, duloxetine 40mg/day in the 1^st^ week, 60mg/day from the 2^nd^ week; VRT not specified.	60 (23/37)	58.66 ± 4.51	duloxetine same as experimental group	8-week	N/R	DHI HAMA HAMD VSI	N/R
Liu 2024 China (20)	PPPD	RCT	31 (18/13)	44.38 ± 9.23	betahistine mesylate+VRT, betahistine mesylate 6mg/time, 3 times/day; VRT not specified.	31 (17/14)	44.13 ± 10.82	betamethasone mesylate same as experimental group	2-month	N/R	VSI BBS	6 cases

VRT, Vestibular rehabilitation training; SRT, specific rehabilitation training; RRT, routine rehabilitation training; NCC, notonginseng & cinnarizine capsule; CBT, cognitive behavioral therapy; TES, transcutaneous electrical stimulation; AGT, acetagastrodin tablets; VDT, visual desensitization therapy; tDCS, Transcranial Direct Current Stimulation; DHI, Dizziness Handicap Inventory; HAMA, Hamilton Anxiety Scale; HAMD, Hamilton Depression Scale; HADS, Hospital Anxiety and Depression Scale; VSI, Vestibular Symptom Index; DSI, indicates Dizziness Symptoms Inventory; SBI, Safety Behaviours Inventory; ABC, Activities-specific Balance Confidence; HDRS, Hamilton Depression Rating Scale; HARS, Hamilton Anxiety Rating Scale; EQ5D, EuroQOL Group quality of life questionnaire; PSQI, Pittsburgh Sleep Quality Index; BBS, Berg Balance Scale; MoCA, Montreal Cognitive Assessment; SAS, Self-Rating Anxiety Scale; SDS, Self-Rating Depression Scale; SSS, Somatization Self-rating Scale.

VRT, Vestibular rehabilitation training; SRT, specific rehabilitation training; RRT, routine rehabilitation training; NCC, notonginseng & cinnarizine capsule; CBT, cognitive behavioral therapy; TES, transcutaneous electrical stimulation; AGT, acetagastrodin tablets; VDT, visual desensitization therapy; tDCS, Transcranial Direct Current Stimulation; DHI, Dizziness Handicap Inventory; HAMA, Hamilton Anxiety Scale; HAMD, Hamilton Depression Scale; HADS, Hospital Anxiety and Depression Scale; VSI, Vestibular Symptom Index; DSI, indicates Dizziness Symptoms Inventory; SBI, Safety Behaviours Inventory; ABC, Activities-specific Balance Confidence; HDRS, Hamilton Depression Rating Scale; HARS, Hamilton Anxiety Rating Scale; EQ5D, EuroQOL Group quality of life questionnaire; PSQI, Pittsburgh Sleep Quality Index; BBS, Berg Balance Scale; MoCA, Montreal Cognitive Assessment. SAS, Self-Rating Anxiety Scale; SDS, Self-Rating Depression Scale; SSS, Somatization Self-rating Scale.

### 3.3 Risk of bias assessment

**Figure 2:**
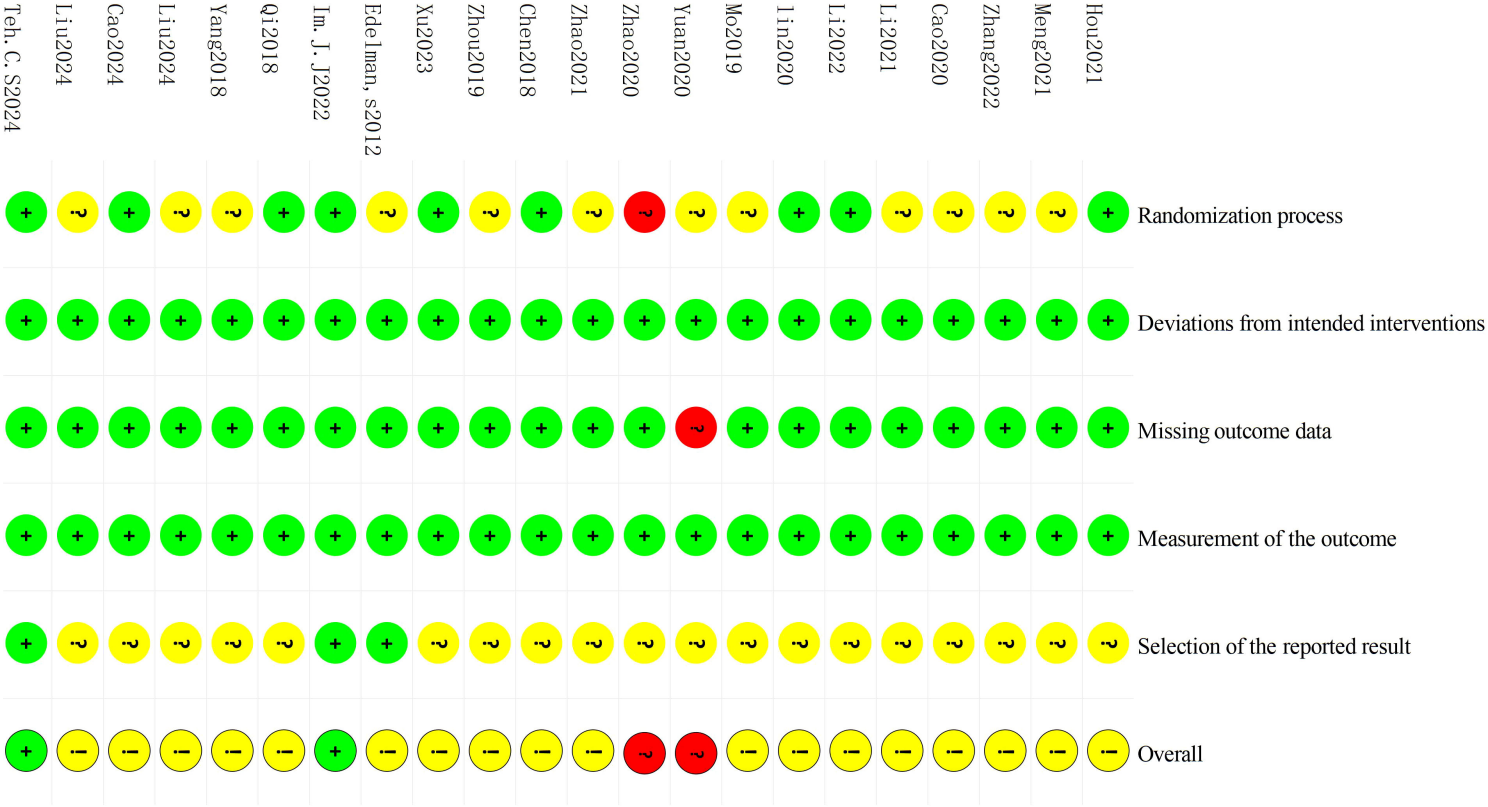
The risk of bias assessment for individual study.

**Figure 3:**
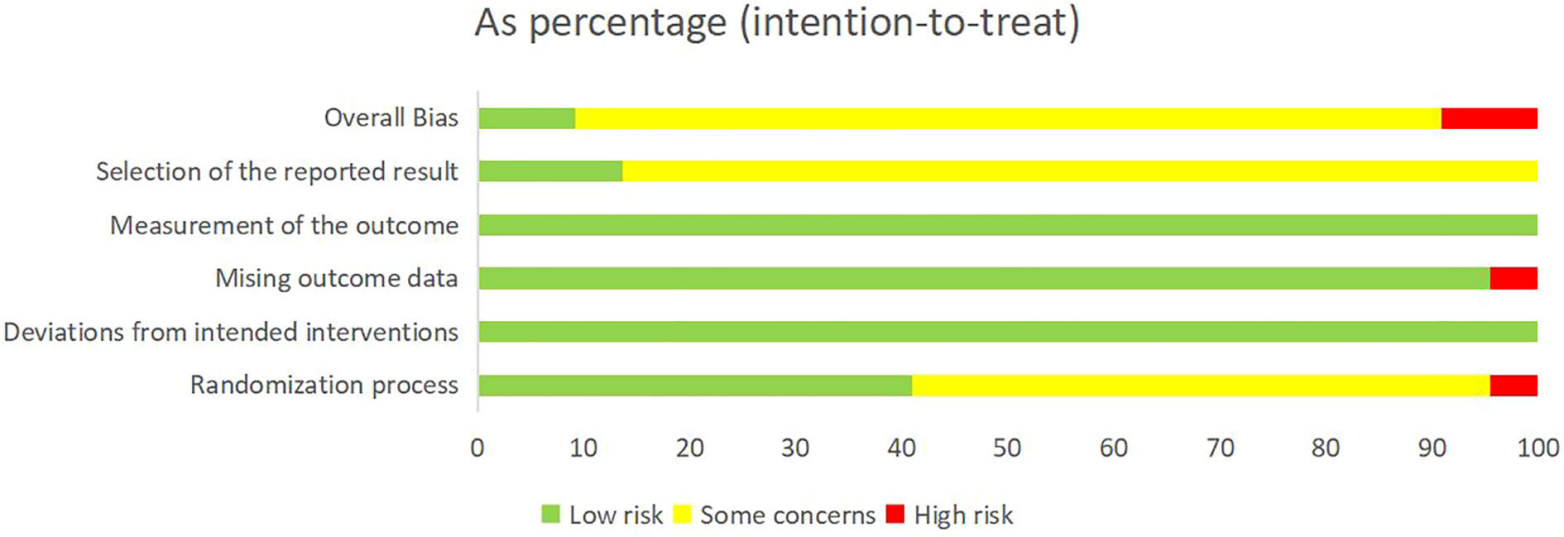
The risk of bias summary.

Cochrane RoB 2 was adopted to assess the risk of bias of the 22 included studies. For the randomization process, 1 study (11) was judged as high risk due to using visit sequence for participant allocation, while 9 studies (9, 13, 15, 16, 19, 21, 23, 25, 27) employing random number tables or computer-generated sequences with adequate allocation concealment were rated as low risk. The remaining studies only mentioned “randomization” without specifying methods, which were rated as some concerns. All studies demonstrated low risk for deviations from intended interventions as no protocol violations were reported. Regarding missing outcome data, 1 study (18) was rated as high risk due to incomplete post-treatment data in the control group without intention-to-treat (ITT) analysis, while the remaining studies were classified as low risk with complete data and loss to follow-up rates ≤10%. All studies employed standardized assessment scales (e.g. DHI, HAMA, etc.) with established reliability and validity, warranting a low risk of bias rating. For outcome reporting, 3 studies (23, 25, 26) demonstrated low risk of bias as their reported results were consistent with registered protocol, while the remaining studies were deemed some concerns due to lack of pre-registered protocols. Detailed results of risk of bias assessment are presented in **Figure 2** and **Figure 3**; **Supplementary File 3**.

Four outcome measures were included in the GRADE assessment for SSRI. For DHI score, 8 RCT were included and the evidence was rated as low (downgraded due to serious risk of bias and inconsistency). For HAMA and HAMD scores, the evidences were rated as low, respectively, (downgraded due to serious risk of bias and inconsistency). For HADS, the evidences was assessed as very low (downgraded for serious risk of bias, inconsistency and imprecision). Three outcome measures were included in the GRADE assessment for VRT. For DHI and HAMD scores, the evidences were rated as moderate, respectively (downgraded due to serious risk of bias). For HAMA score, the evidence was rated as low (downgraded due to serious risk of bias and inconsistency). (**Supplementary File 4** – GRADE).

### 3.4 Result of comparisons of the effect of different conservative interventions for PPPD

#### 3.4.1 Selective serotonin reuptake inhibitors

Due to high heterogeneity of interventions using SSRI across included studies, we divided these studies into three types: SSRI directly compared with other conservative therapies, SSRI plus other conservative therapies compared with SSRI alone, and one type of SSRIs compared with another type of SSRIs combined with other conservative therapies or not.

One study (10) compared citalopram directly with vestibular rehabilitation training (VRT) for persistent posture-perceptual dizziness. The citalopram group demonstrated a greater improvement on DHI scores at 30 days from baseline (mean change 24.38 ± 8.08), compared with VRT group (16.53 ± 9.92), the between-group difference (MD 7.85, 95%CI4.12, 11.58; p<0.05) was significant. The difference of HAMA scores was also significant between the two groups (MD 1.7, 95%CI0.33, 3.07; p<0.05). For HAMD scores, citalopram (5.5 ± 2.33) showed significantly greater improvement compared with VRT (2.66 ± 2.39), with a between-group difference (MD 2.84, 95%CI1.50,4.18; p<0.05). (**Table 2**).

**Table 2:** Outcome measures of included studies.

Author Year	Sample size	Outcome measurement, mean ± SD	Experiment group			Control group			Difference of changes	
			Baseline	After treatment	Changes	Baseline	After treatment	Changes	MD	P-value
Hou 2021 (21)	43/43	DHI	63.67 ± 6.1	34.77 ± 3.95	28.9 ± 5.79	62.94 ± 5.9	46.42 ± 4.22	16.52 ± 5.72	12.38 ± 6.3	P<0.05
		HAMA	18.63 ± 2.41	8.14 ± 2.08	10.49 ± 2.47	18.78 ± 3.16	14.45 ± 2.67	4.33 ± 3.22	6.16 ± 3.18	P<0.05
		HAMD	20.82 ± 3.23	9.51 ± 1.54	11.31 ± 2.97	20.75 ± 3.16	15.8 ± 2.78	4.95 ± 3.27	6.36 ± 3.43	P<0.05
Meng 2021 (22)	71/71	DHI	53.25 ± 7.43	26.08 ± 4.12	27.17 ± 6.91	54.78 ± 6.45	50.01 ± 3.62	4.77 ± 6	22.4 ± 7.11	P<0.05
		VSI	37.82 ± 3.42	12.89 ± 3.64	24.93 ± 3.87	36.92 ± 3.72	19.01 ± 2.34	17.91 ± 3.51	7.02 ± 4.06	P<0.05
Zhang 2022 (6)	45/45	SAS	56.79 ± 4.41	44.3 ± 4.13	12.49 ± 4.68	56.1 ± 4.39	51.15 ± 4.19	4.95 ± 4.7	7.54 ± 5.14	P<0.05
		SDS	55.79 ± 4.21	38.3 ± 4.11	17.49 ± 4.56	55.1 ± 4.19	49.15 ± 4.2	5.95 ± 4.6	11.54 ± 5.01	P<0.05
Cao 2020 (7)	30/30	DHI	53.33 ± 20.57	19.33 ± 10.24	34 ± 18.96	53.53 ± 21.22	30.97 ± 15.13	22.56 ± 20.55	11.44 ± 21.68	P<0.05
		HADS	15.47 ± 6.73	4.43 ± 3.27	11.04 ± 6.2	15.8 ± 7.3	8.13 ± 6.42	7.67 ± 7.55	3.37 ± 7.61	P<0.05
Li 2021 (8)	30/30	DHI	58.18 ± 15.02	39.01 ± 6.18	19.17 ± 13.77	59.89 ± 15	46.15 ± 4.25	13.74 ± 13.86	5.43 ± 15.13	P<0.05
Li 2022 (27)	40/40	SSS	40.62 ± 8.79	15.16 ± 2.23	25.46 ± 8.16	40.31 ± 9.14	22.08 ± 4.31	18.23 ± 8.4	7.23 ± 9.07	P<0.05
		HAMA	18.96 ± 2.09	12.34 ± 2.17	6.62 ± 2.33	19.47 ± 2.28	16.35 ± 2.12	3.12 ± 2.41	3.5 ± 2.6	P<0.05
Lin 2020 (9)	29/29	DHI	58.2 ± 3	27.72 ± 2.84	30.48 ± 3.2	57.75 ± 2.79	36.89 ± 3.03	20.86 ± 3.19	9.62 ± 3.5	P<0.05
		HADS	14.62 ± 2.29	4.24 ± 1.35	10.38 ± 2.14	15.31 ± 2.58	6.03 ± 1.74	9.28 ± 2.47	1.1 ± 2.54	P<0.05
Mo 2019 (10)	70/70	DHI	54.62 ± 8.51	30.24 ± 5.52	24.38 ± 8.08	55.05 ± 9.65	38.52 ± 8.36	16.53 ± 9.92	7.85 ± 9.98	P<0.05
		HAMA	16.62 ± 2.11	13.54 ± 2.19	3.08 ± 2.36	16.59 ± 2.25	15.21 ± 2.27	1.38 ± 2.48	1.7 ± 2.65	P<0.05
		HAMD	18.85 ± 2.13	13.35 ± 2.12	5.5 ± 2.33	19.03 ± 2.22	16.37 ± 2.15	2.66 ± 2.39	2.84 ± 2.59	P<0.05
Yuan 2020 (18)	33/30	DHI	32.18 ± 6.92	25.52 ± 9.86	6.66 ± 9.51	38.16 ± 11.17	32.36 ± 8.98	5.8 ± 11.19	0.86 ± 11.43	P>0.05
Zhao 2020 (11)	29/27	DHI	43.93 ± 9.22	0.34 ± 0.77	43.59 ± 8.94	43.11 ± 8.86	3.33 ± 1.24	39.78 ± 8.44	3.81 ± 9.53	P<0.05
		HADS	20.14 ± 4.09	0.38 ± 0.38	19.76 ± 3.95	8.89 ± 3.48	1.74 ± 0.74	7.15 ± 3.25	12.61 ± 3.99	P<0.05
Zhao 2021 (12)	29/29	DHI	65.73 ± 9.73	30.43 ± 9.67	35.3 ± 10.63	65.74 ± 9.52	44.98 ± 9.62	20.76 ± 10.48	14.54 ± 11.56	P<0.05
		HAMA	20.27 ± 3.13	7.22 ± 3.37	13.05 ± 3.57	20.14 ± 3.37	13.53 ± 3.37	6.61 ± 3.69	6.44 ± 3.98	P<0.05
		HAMD	21.93 ± 3.35	9.48 ± 3.71	12.45 ± 3.88	21.75 ± 3.29	14.56 ± 3.27	7.19 ± 3.59	5.26 ± 4.1	P<0.05
Chen 2018 (13)	75/75	DHI	54.3 ± 4.5	22.6 ± 4.1	31.7 ± 4.72	54.2 ± 4.4	28.3 ± 4.2	25.9 ± 4.71	5.8 ± 5.17	P<0.05
		HAMA	17.5 ± 2.6	10.4 ± 1.9	7.1 ± 2.53	17.4 ± 2.7	12.3 ± 1.8	5.1 ± 2.58	2 ± 2.8	P<0.05
		HAMD	18.4 ± 2.3	11.1 ± 2.2	7.3 ± 2.37	18.3 ± 2.2	13.6 ± 1.9	4.7 ± 2.26	2.6 ± 2.54	P<0.05
Zhou 2019 (14)	32/32	DHI	64.93 ± 7.38	29.05 ± 4.2	35.88 ± 6.88	65.19 ± 8.12	40.14 ± 5.68	25.05 ± 7.83	10.83 ± 8.09	P<0.05
Xu 2023 (19)	30/30	DHI	71.92 ± 8.72	12.67 ± 5.46	59.25 ± 8.23	71.8 ± 6.86	18.83 ± 5.72	52.97 ± 6.96	6.28 ± 8.39	P<0.05
		VSI	7.2 ± 0.98	1.33 ± 0.56	5.87 ± 0.91	7.17 ± 0.64	1.92 ± 0.5	5.25 ± 0.64	0.62 ± 0.88	P<0.05
Edelman, S 2012 (26)	20/21	DHI	53.8 ± 20.38	26.8 ± 18.7	27 ± 21.45	34.76 ± 20.73	16.48 ± 14.41	18.28 ± 19.96	8.72 ± 22.72	P<0.05
		DSI	26.5 ± 12.21	14.2 ± 8.49	12.3 ± 11.76	19.01 ± 10.92	10.48 ± 10.48	8.53 ± 11.73	3.77 ± 12.86	P<0.05
		SBI	31.15 ± 14.45	12.2 ± 8.92	18.95 ± 13.61	18.86 ± 12.38	6.19 ± 5.13	12.67 ± 11.35	6.28 ± 13.8	P<0.05
Im, J. J. 2022 (25)	12/11	DHI	34.3 ± 15.9	25.1 ± 13.6	9.2 ± 16.27	35.3 ± 14.2	19.1 ± 14.3	16.2 ± 15.61	‘-7 ± 17.47	P=0.79
		ABC	77.3 ± 21	75.34 ± 24.7	1.96 ± 25.22	77.6 ± 17.5	85 ± 15.7	‘-7.4 ± 17.97	9.36 ± 24.42	P=0.45
		HDRS	5.4 ± 3.2	5.3 ± 3.59	0.1 ± 3.73	5.8 ± 5.6	9.73 ± 6.8	‘-3.93 ± 6.87	4.03 ± 6.37	P=0.60
		HARS	6.6 ± 3.2	6.09 ± 4.23	0.51 ± 4.16	7.6 ± 6.4	8.91 ± 7.65	‘-1.31 ± 7.77	1.82 ± 7.2	P=0.47
Teh, C. S 2024 (23)	30/29	DHI	48.2 ± 22.5	25.3 ± 18.9	22.9 ± 2.87	52.9 ± 22.7	37.9 ± 24.3	15 ± 25.78	7.9 ± 26.76	P=0.0003
		EQ5D	70.8 ± 18.9	81.2 ± 12.4	‘-10.4 ± 17.99	66.4 ± 16.9	11.4 ± 7.2	55 ± 15.5	‘-65.4 ± 18.76	P=0.16
Qi 2018 (15)	60/60	Anxiety score	7.88 ± 1.73	2.11 ± 0.56	5.77 ± 1.59	7.93 ± 1.11	3.78 ± 0.67	4.15 ± 1.04	1.62 ± 1.51	P<0.05
Yang, 2018 (24)	48/48	DHI	63.52 ± 10.97	28.46 ± 12.03	35.06 ± 12.63	68.35 ± 10.79	42.19 ± 12.06	26.16 ± 12.56	8.9 ± 13.8	P<0.05
		HAMA	19.87 ± 4.01	7.98 ± 3.99	11.89 ± 4.38	19.77 ± 3.78	13.21 ± 4.07	6.56 ± 4.31	5.33 ± 4.76	P<0.05
		HAMD	20.87 ± 4.22	8.77 ± 2.41	12.1 ± 3.93	20.95 ± 3.99	15.21 ± 3.63	5.74 ± 4.18	6.36 ± 4.45	P<0.05
Cao 2024 (16)	37/37	DHI	54.59 ± 5.98	11.52 ± 3.54	43.05 ± 5.6	54.43 ± 5.67	23.34 ± 4.16	31.09 ± 5.53	11.96 ± 6.1	P<0.05
		HADS	17.80 ± 2.95	6.64 ± 1.28	11.16 ± 2.71	17.68 ± 3.03	11.57 ± 1.90	6.11 ± 2.86	5.05 ± 3.05	P<0.05
		PSQI	14.14 ± 1.43	7.05 ± 1.12	7.09 ± 1.42	13.97 ± 1.38	10.78 ± 1.25	3.19 ± 1.44	3.9 ± 1.57	P<0.05
		BBS	29.32 ± 3.89	47.90 ± 5.78	‘-18.58 ± 5.53	29.41 ± 3.94	38.27 ± 4.61	‘-8.86 ± 4.72	‘-9.72 ± 5.65	P<0.05
		MoCA	18.94 ± 3.12	28.69 ± 1.67	‘-9.75 ± 2.89	19.25 ± 3.07	24.88 ± 2.15	‘-5.63 ± 2.96	‘-4.12 ± 3.21	P<0.05
Liu 2024 (17)	60/60	DHI	72.02 ± 5.19	12.03 ± 3.24	59.99 ± 4.9	72.26 ± 5.66	19.75 ± 4.55	54.31 ± 5.67	5.68 ± 5.82	P<0.05
		HAMA	14.31 ± 3.27	4.58 ± 1.80	9.73 ± 3.04	14.28 ± 3.46	7.32 ± 1.95	6.96 ± 3.22	2.77 ± 3.43	P<0.05
		HAMD	13.22 ± 3.09	3.24 ± 1.16	9.98 ± 2.83	13.37 ± 3.14	6.55 ± 1.20	6.82 ± 2.88	3.16 ± 3.13	P<0.05
		VSI	7.15 ± 0.59	1.23 ± 0.23	5.92 ± 0.54	7.17 ± 0.46	2.35 ± 0.31	4.82 ± 0.44	1.1 ± 0.54	P<0.05
Liu 2024 (20)	31/31	VSI	45.15 ± 5.29	23.54 ± 3.12	21.61 ± 4.95	46.25 ± 5.24	29.64 ± 3.76	16.61 ± 5.08	5 ± 5.5	P<0.05
		BBS	18.28 ± 2.29	46.28 ± 4.29	‘-28 ± 4.97	18.59 ± 2.27	41.39 ± 4.58	‘-22.8 ± 4.42	‘-5.2 ± 4.49	P<0.05

Ten studies compared SSRI plus other conservative therapies with the same SSRI used alone. Among these, 3 studies (6, 8, 11) with 206 patients were on sertraline, 4 studies (12–15) with 392 patients on citalopram, 1 study (9) with 58 patients on fluoxetine, and 2 studies (16, 17) with 194 patients on duloxetine. Due to the comparable underlying mechanism and interventions design, these studies were analyzed and reported together by SSRI type and outcome measurements:

(1) Dizziness Handicap Inventory (DHI)

Eight studies (8, 9, 11–14, 16, 17) reported DHI scores as outcome measure. Three studies on citalopram (12–14) all found that citalopram plus conservative therapy group (VRT and psychological therapy, acupuncture and biofeedback-CBT, respectively) had greater reductions on DHI scores compared with citalopram alone group (all p<0.05). The pooled estimate of the mean difference of DHI score between two groups in the 3 studies was 9.90 (95%CI 4.78,15.02; p=0.0002), which was in favor of citalopram plus conservative therapy group and consistent with individual study.

Two studies on sertraline (8, 11) reported that the change of DHI score after treatment was comparable in sertraline plus conservative therapy group (acupuncture and visual desensitization therapy, respectively) and sertraline alone group (p>0.05), however, the pooled estimate of the mean difference of DHI score between two groups in the 2 studies was 4.29 (95%CI 0.48,8.11; p=0.03), which was not consistent with individual study.

Two studies on duloxetine (16, 17) also reported significantly greater improvements on DHI score in duloxetine plus VRT group compared with duloxetine alone group (p<0.05). The pooled estimate of the mean difference of DHI score between two groups in the 2 studies was 8.76 (95%CI 2.61,14.91; p=0.005), which was in favor of duloxetine plus VRT group.

One study compared fluoxetine plus transcranial electrical stimulation (TES) with fluoxetine alone (9) and it showed the improvement of DHI score after treatment was greater in fluoxetine plus TES group (p<0.05) (**Table 2**).

**Figure 4:**
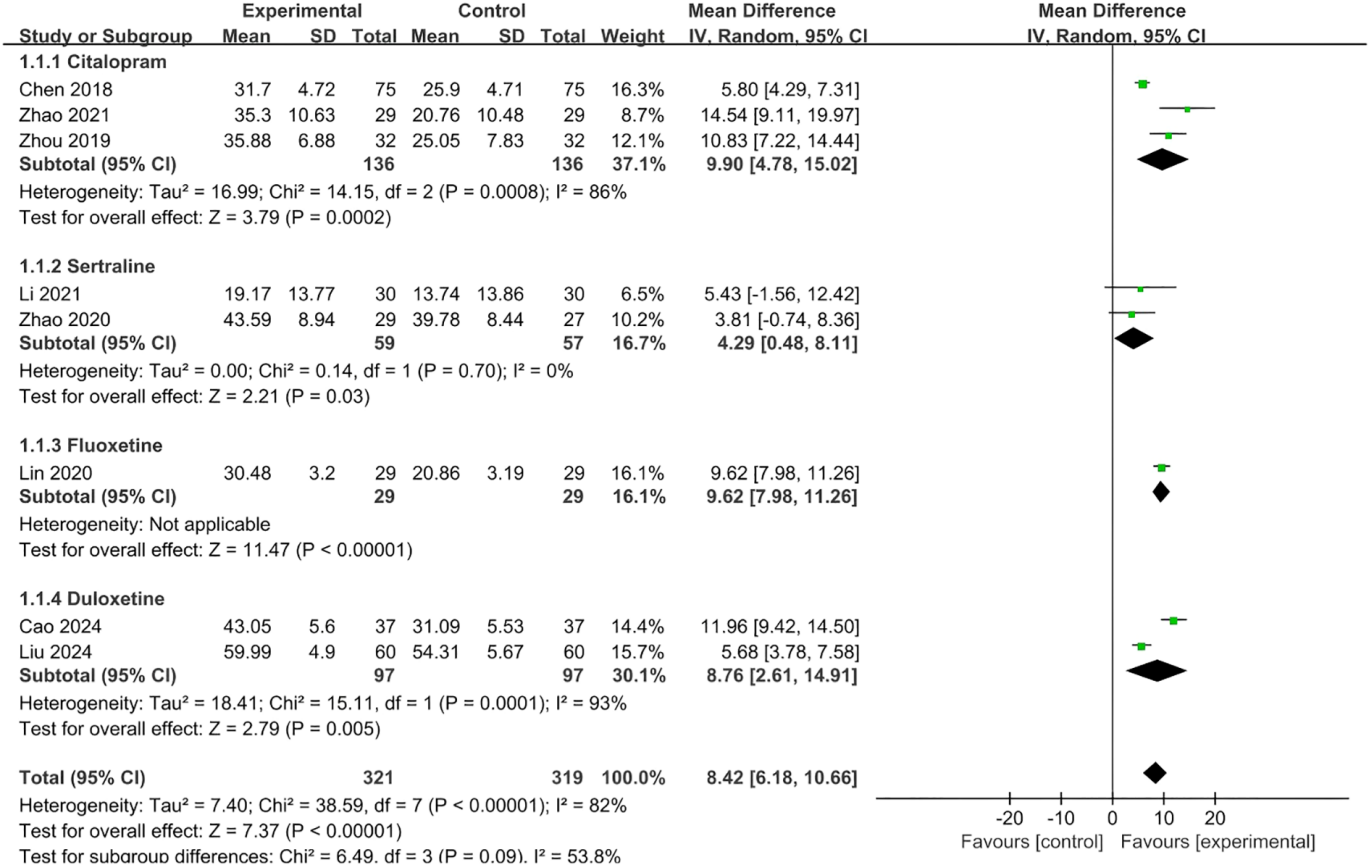
Meta-analysis of effect of SSRI plus conservative therapies versus SSRI alone on DHI scores for PPPD patients.

The overall pooled estimate of the mean difference of DHI score between two groups across 8 studies was 8.42 (95%CI 6.18,10.66; p<0.001), which was in favor of SSRI (citalopram/sertraline/duloxetine/fluoxetine) plus conservative therapy groups. Due to substantial heterogeneity among interventions, a random-effects model was employed (**Figure 4**).

The sensitivity analyses were conducted by moving one type of SSRI at a time, and the result showed that the direction of the overall pooled result was not change (pooled mean difference of DHI ranged from 7.78 to 9.23, all p<0.05), which indicated the certainty of the pooled result of DHI (**Supplementary File 5** - Sensitivity analyses of DHI for SSRI).

(2) HAMA scores

**Figure 5:**
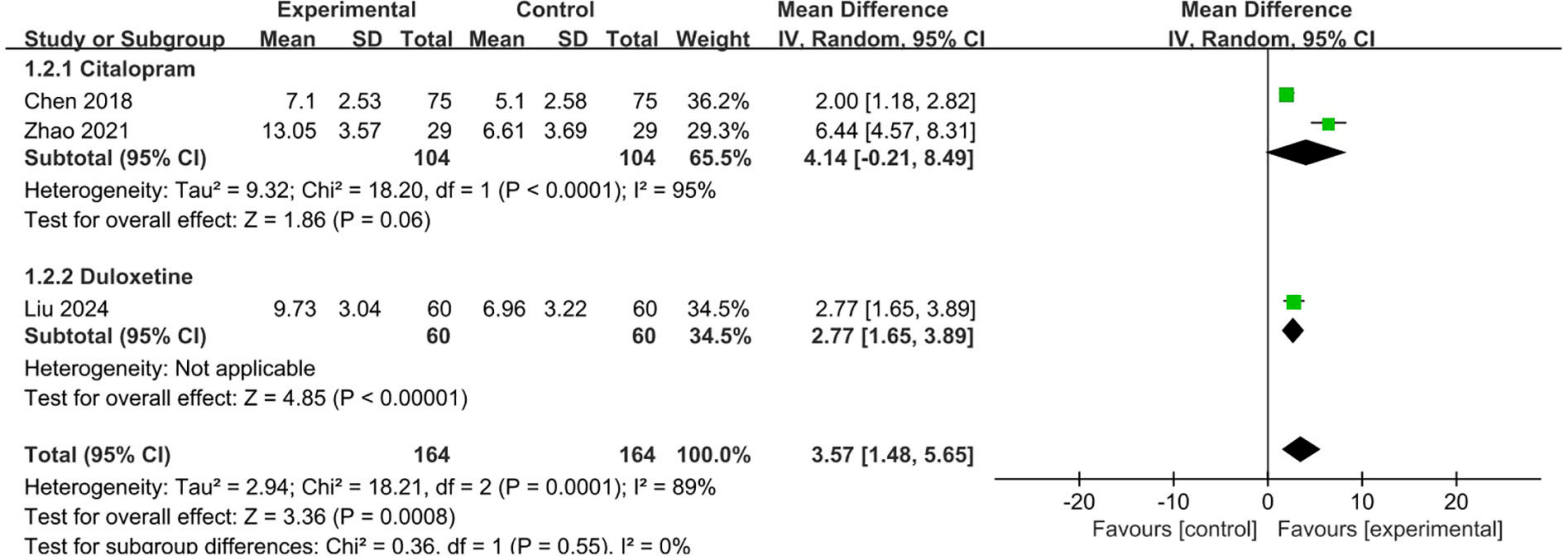
Meta-analysis of SSRI combined with conservative therapies versus SSRI alone on HAMA scores for PPPD patients.

Three studies (12, 13, 17) with 328 patients reported HAMA scores, among which two compared citalopram plus conservative therapies (VRT plus psychological therapy or acupuncture, respectively) with citalopram alone, and one compared duloxetine plus VTR with duloxetine alone. The pooled estimate of the mean difference of HAMA score between two groups in the 3 studies was 3.57 (95%CI 1.48,5.65; p=0.0008), which was in favor of SSRI plus conservative therapy group and consistent with individual study. Due to substantial heterogeneity (I^2^ = 89%), a random-effects model was employed (**Figure 5**).

The sensitivity analyses were conducted by moving one type of SSRI at a time. When the duloxetine group (17) was removed, the pooled mean difference of HAMA was 4.14 (95%CI -0.21,8.49; p=0.06), which indicated the uncertainty of the result of HAMA. (**Supplementary Figure 1**).

(3) HAMD scores

**Figure 6:**
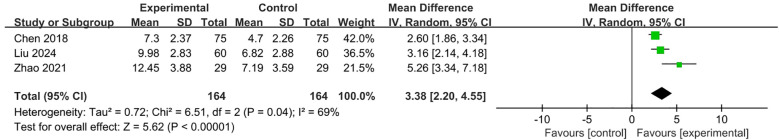
Meta-analysis of SSRI combined with conservative therapies versus SSRI alone on HAMD scores for PPPD patients.

Above mentioned 3 studies (12, 13, 17) also reported HAMD scores. Given moderate heterogeneity (I²=69%), a random-effects model was applied. The pooled estimate of the mean difference of HAMD score between two groups in the 3 studies was 3.38 (95%CI 2.20,4.55; p<0.0001), which was in favor of SSRI plus conservative therapy group and consistent with individual study (**Figure 6**).

(4) HADS scores

**Figure 7:**
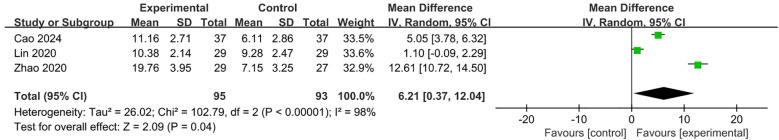
Meta-analysis of SSRI combined with conservative therapies versus SSRI alone on HADS scores for PPPD patients.

Three studies (9, 11, 16) with 188 patients evaluated HADS scores, among which one compared fluoxetine plus TES versus fluoxetine alone, one compared sertraline plus VDT versus sertraline alone, one compared duloxetine plus VRT versus duloxetine alone. Due to substantial heterogeneity across interventions (I²=98%), a random-effects model was used. The pooled estimate of the mean difference of HADS score between two groups in the 3 studies was 6.21 (95% CI 0.37,12.04; p=0.04), which was in favor of SSRI plus conservative therapy group (**Figure 7**).

One study (7) compared duloxetine with escitalopram with conservative therapies (biofeedback-CBT plus VRT) in both groups at the same time for PPPD patients, showing that the duloxetine group had a greater improvement on DHI score after 6-week treatment from baseline (mean change 34 ± 18.96), compared with escitalopram group (mean change 22.56 ± 20.55), with a between-group difference of 11.44(95%CI0.23, 22.65; p<0.05). The for HADS scores, the duloxetine group also demonstrated a great change on HADS score (mean change 11.04 ± 6.2) compared with escitalopram group (mean change 7.67 ± 7.55), with no significant between-group difference of 3.37 (95%CI (-0.57,7.31); P>0.05). Due to substantial heterogeneity across interventions, this study was not included in the meta-analysis. Another study (15) compared citalopram plus conservative therapies (VRT plus CBT, respectively) with citalopram alone, showing the combined therapy had a greater improvement on anxiety score after 6-week treatment from baseline, compared with escitalopram group, with a between-group difference of 1.62 (95%CI 1.1,2.2; p<0.05). (**Table 2**).

#### 3.4.2 Other medications

**Figure 8:**
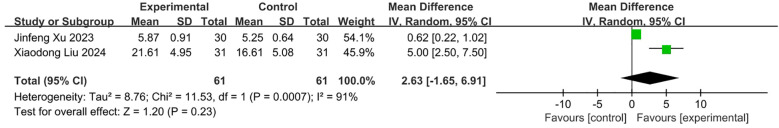
Meta-analysis of betahistine plus conservative interventions versus betahistine alone on VSI scores for PPPD patients.

Two Chinese studies (19, 20) compared betahistine tablets plus VRT with betahistine alone for PPPD patients. Both studies reported on Vestibular Symptom Index (VSI). The pooled estimate of the mean difference of VSI score between two groups in the 2 studies was 2.63 (95% CI -1.65,6.91; p=0.23), which was not significant (**Figure 8**).

One Chinese study (18) compared deanxit combined with acetazolamide tablets versus deanxit alone for PPPD. It showed that the combined treatment had a greater improvement on DHI compared with deanxit alone at 2 weeks from baseline, with a between-group difference of 0.86 (95%CI -4.91,6.63; p>0.05).

#### 3.4.3 Vestibular rehabilitation training

Five studies (21–24, 27) with 434 patients were included in this category. Three studies (21, 24, 27) with 262 patients compared VRT plus other conservative therapies with VRT alone for PPPD. One study (22) with 142 patients compared specialized VRT with routine VRT on the basis of medication in both groups. Another study (23) with 59 patients compared home-based VRT with hospital-based VRT for PPPD management (**Table 1**).

(1) DHI scores

**Figure 9:**
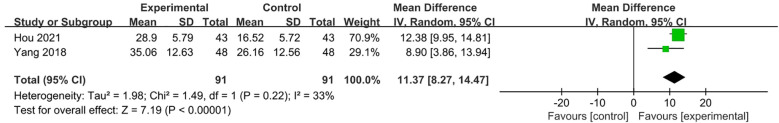
Meta-analysis of VRT plus SSRI versus VRT alone on DHI scores for PPPD patients.

Four studies (21–24) with 383 patients reported DHI scores as outcome measure. Two studies (21, 24) comparing VRT plus SSRI (with CBT added in one study) with VRT were included in the meta-analysis. Due to heterogeneity observed in intervention design (e.g. dose of drugs) and duration (e.g. 4 or 8 weeks), a random-effects model was used and the pooled estimate of the mean difference of DHI score between two groups in the 2 studies was 11.37 (95% CI 8.27,14.47; p<0.0001), which was in favor of the combined treatment group and consistent with individual study (**Figure 9**).

In addition, one study (22) showed that after one month of treatment, specialized VRT led to a greater reduction on DHI compared with routine VRT, with a between-group difference of 22.4 (95%CI20.1,24.7; p<0.05) both on the basis of medication in both groups. The other study (23) showed that both home-based VRT and hospital-based VRT made significantly greater improvement on DHI at 12 weeks from baseline, however, the between-group difference (7.9 95%CI-6.1,21.9; p=0.16) was not significant in this study (**Table 2**).

(2) HAMA scores

**Figure 10:**
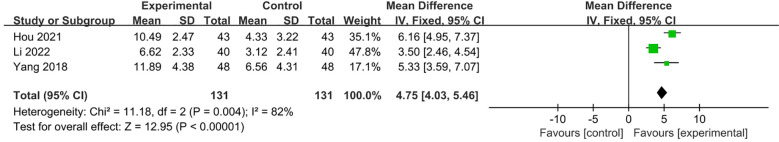
Meta-analysis of VRT plus SSIR and/or other conservative therapy with VRT alone on HAMA scores for PPPD patients.

Three studies (21, 24, 27) with 262 patients reported HAMA scores, all comparing VRT plus SSIR and/or other conservative therapy with VRT alone. Due to significant heterogeneity among interventions (I²=82%), a random-effects model was employed. The pooled estimate of the mean difference of HAMA score between two groups in the 3 studies was 4.75 (95% CI 4.03,5.46; p<0.0001), which was in favor of the combined treatment group and consistent with individual study (**Figure 10**).

(3) HAMD scores

**Figure 11:**
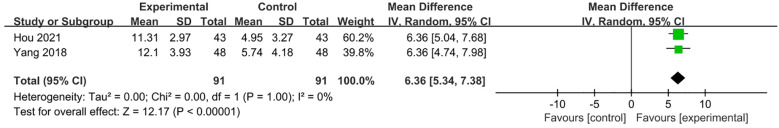
Meta-analysis of VRT plus SSIR and/or other conservative therapy with VRT alone on HAMD scores for PPPD patients.

Two studies (21, 24) with 182 patients reported HAMD scores. The pooled estimate of the mean difference of HAMD score between two groups in the 2 studies was 6.36 (95% CI 5.34, 7.38; p<0.0001), which was in favor of the combined treatment group (**Figure 11**).

In addition, one study (22) reported VSI, showing that the experiment group had significant improvement on VSI compared with control group. Another study (23) showed that improvement on EQ-5D in the experiment group were not significantly great than that in the control group (**Table 2**).

#### 3.4.4 Transcranial direct current stimulation

Only one study (25) compared tDCS with sham tDCS for patients with PPPD. It reported that both groups made improvement on outcome measure of DHI, Activities-specific Balance Confidence (ABC), Hamilton Depression Rating Scale (HDRS), and Hamilton Anxiety Rating Scale (HARS) scores, however, the between-group differences on these outcomes were not significant (all p>0.05) (**Table 2**).

#### 3.4.5 Cognitive behavioral therapy

One study (26) compared CBT with wait-list control for patients with chronic subjective dizziness. It reported that both groups made improvement on outcome measure of Dizziness Symptoms Inventory (DSI), DHI, Safety Behaviors Inventory (SBI) after 3-week treatment, and the between-group differences on these outcomes were significant for all. (**Table 2**).

## 4 Discussion

This systematic review and meta-analysis synthesized evidence from 22 RCTs (1,764 patients) to evaluate the effect of conservative therapies in promoting functional recovery and symptom alleviation for PPPD. For SSRI and VRT, the pooled estimates presented consistent results that the combined therapy had significant improvements on DHI and HAMA compared with single therapy. However, the certainty of the effect of CBT and tDCS were unclear due to limited number of studies and small sample size. The major concern of risk of bias of included studies laid to selection of reported results and randomization process.

SSRIs have been widely validated as first-line medications for PPPD. It modulate the serotonin (5-HT) system, which could influence postural control through brainstem-cerebellar-vestibular pathways (28). This review found that SSRI drugs (e.g., sertraline, citalopram) combined with VRT or CBT demonstrated significantly improvement on dizziness symptom using DHI scores and anxiety/depressive symptoms using HAMA/HAMD/HADS compared with single therapy. The eight studies included in the meta-analysis of DHI showed that the improvement of DHI score in both experimental and control groups were higher than the MCID of 11 points (29), however, the pooled between-group difference of DHI (MD 8.42, 95% CI 6.18, 10.66; p<0.001) did not exceed the MCID (**Figure 4**), and same trends were observed in sensitivity analysis by removing one type of SSRI at a time. The observed between-group difference in HAMA scores (**Figure 5**, MD 3.57, 95% CI 1.48, 5.65; p=0.0008) exceeded the established MCID of 2.5 points (30), as well as the change of HAMA score in both experimental and control groups in the three studies for meta-analysis. For HADS scores, although its pooled result of between-group difference (**Figure 7**, MD 6.21, 95%CI 0.37,12.04; p=0.04) exceeded its MCID of approximately 5.7 points (31), only one out of three individual studies in the meta-analysis generated similar result. Considering the heterogeneity across intervention design of included studies (i.e. type of SSRI and combined therapies), the pooled result may exaggerate the actual treatment effect of individual type of intervention. In terms of HAMD, as its MCID is highly context-dependent and often reported as a percentage reduction (e.g., 50% from baseline) rather than a fixed value, it is hard to make a direct comparison. The pooled between-group difference of HAMD in this study was statistically significant in favor of experimental group and consistent with other outcome measures, however, due to the heterogeneity across the three included studies, the result needs to be interpreted with caution. Majority of the studies on SSRI were rated as some concerns in risk of bias assessment due to unclear description of selection of the reported results and randomization process, and one study rated as high risk for problematic randomization. And the certainty of evidence for each outcome of SSRI was rate as low to very low in GRADE assessment. Therefore, above mentioned pooled results on DHI, HAMA, HAMD, etc. can be overestimated due to imbalanced characteristics of patients in the two treatment groups and missing outcomes.

In review found that VRT combined with SSRI demonstrated consistent therapeutic efficacy across studies on DHI, HAMA and HAMD compared with VRT alone. The pooled between-group difference of DHI score was higher than its MCID of 11 points (**Figure 9**), and the pooled between-group difference of HAMA score exceeded its MCID of 2.5 points (**Figure 10**). The three studies included in meta-analyses of DHI, HAMA and HAMD, respectively, were all rate some concerns in risk of bias assessment due to unclear description of selection of the reported results, and the certainty of evidence for each outcome of VRT was rated as moderate to low in GRADE assessment. Therefore, the pooled result of these outcome measures should be explained with caution.

Among emerging interventions, CBT demonstrated potential for improving DHI and DSI scores, though current evidence derived from single-center trials with limited sample sizes. CBT primarily works by modifying catastrophic interpretations of dizziness symptoms and gradually reducing avoidance behaviors through exposure therapy (32). However, PPPD patients frequently exhibit alexithymia, which may compromise emotional identification and expression during CBT. Future studies should develop adapted CBT protocols better tailored to PPPD patients’ specific needs to enhance feasibility. Regarding neuromodulation techniques, transcranial direct current stimulation (tDCS) showed no significant efficacy in preliminary studies (25), potentially due to suboptimal target selection (e.g., primary motor cortex versus dorsolateral prefrontal cortex) or parameter settings (current intensity/treatment duration). These findings underscore the need for refined selection of target population and protocol optimization with standardize stimulation parameters (e.g., current density, electrode size, number of sessions, duration, etc.) in non-invasive neuromodulation approaches.

One study reviewing the pharmacological intervention (SSRI and SNRI) for PPPD found no evidence from eligible placebo-controlled randomized trials. And it excluded studies that did not use the Bárány Society criteria to diagnose PPPD and studies that followed up participants for less than three months (4). Therefore, it is not possible to make any direct comparison on key outcomes between this study and our review. One review (33) showed that combining additional CBT with conventional therapy(including VRT, SSRI, SNRI) significantly improved outcomes for PPPD patients (n=416) compared with conventional therapy alone, especially in DHI scores (MD −8.17, 95% CI −10.26, −6.09; p < 0.00001), and HAMA scores (MD −2.76, 95% CI−3.57, −1.94; p < 0.00001). These findings are similar to what we found in this review for SSRI and VRT interventions, which may be a result of using CBT as complementary treatment for these two types of interventions included in our review. Therefore, the effect of combined intervention of SSRI and CBT/VRT is very promising for patient with PPPD and consistent across reviews. However, due to the substantial heterogeneity in interventions design across SSRI, VRT and CBT studies included in the two reviews, the effect size of such combined interventions is inconclusive.

One review (34) on non-pharmacological interventions for patients with PPPD or functional dizziness (n=1362) found that SMDs for DHI of most of trials were between 0.04 and 1.05, which were much lower than that found in this review for VRT, CBT and tDCS. The major reason was likely due to the difference in design of comparison groups for non-pharmacological interventions in the two reviews. Another review specifically on (35) vestibular physical therapy (VPT), similar to VRT in this review, also reported a pooled mean difference of DHI scores (1.60, 95% CI 0.75, 2.45), which is much lower than that found in this study. This may be largely attributable to the unstandardized regimen of vestibular therapy.

Regarding safety outcomes, although no significant between-group differences were observed in the incidence of adverse events across all included studies, only two out of 22 included studies reported such data. This may lead to underrating of the side effect of interventions included in this review. Consequently, patient’s compliance that is closely associated with side effect could be underestimated and overall benefit of the interventions overestimated.

This study has several limitations. Firstly, substantial heterogeneity in intervention regimens among included studies may compromise the robustness of meta-analysis results; Secondly, some studies lacked blinding procedures or detailed randomization descriptions, introducing potential performance bias; Thirdly, most trials did not include long-term follow-up, precluding assessment of treatment durability; Fourthly, the possible incomplete reporting of adverse events may lead to overestimation of overall treatment effect of all types of interventions. Finally, small sample sizes in non-SSRI pharmacotherapy and other interventions (e.g. tDCS, CBT) limit further analysis and the generalizability of findings.

## 5 Conclusion

Conservative therapies, particularly SSRIs combined with VRT or CBT, could improve functional status and symptom severity in PPPD patients with favorable safety profiles. Based on current evidence, we recommend to prioritize SSRI plus structured VRT as treatment option for patient with PPPD. Future multicenter RCTs with larger sample size and standardized outcome measures are needed to further verify the effect of those conservative therapies, especially for CBT and tDCS, to inform clinical decision-making.

## Data Availability

The raw data supporting the conclusions of this article will be made available by the authors, without undue reservation.

## References

[1] Staab JP Eckhardt-Henn A Horii A Jacob R Strupp M Brandt T . Diagnostic criteria for persistent postural-perceptual dizziness (PPPD): Consensus document of the committee for the Classification of Vestibular Disorders of the Bárány Society. J Vestib Res. (2017) 27:191–208. doi: 10.3233/VES-170622, PMID: 29036855 PMC9249299

[2] Fung KW Xu J Bodenreider O . The new International Classification of Diseases 11th edition: a comparative analysis with ICD-10 and ICD-10-CM. J Am Med Inform Assoc. (2020) 27:738–46. doi: 10.1093/jamia/ocaa030, PMID: 32364236 PMC7309235

[3] Staab JP . Persistent postural-perceptual dizziness. Semin Neurol. (2020) 40:130–7. doi: 10.1055/s-0039-3402736, PMID: 31935771

[4] Webster KE Harrington-Benton NA Judd O Kaski D Maarsingh OR MacKeith S . Pharmacological interventions for persistent postural-perceptual dizziness (PPPD). Cochrane Database Syst Rev. (2023) 3:CD015188. doi: 10.1002/14651858.CD015188 36906836 PMC9997546

[5] Axer H Finn S Wassermann A Guntinas-Lichius O Klingner CM Witte OW . Multimodal treatment of persistent postural-perceptual dizziness. Brain Behav. (2020) 10:e01864. doi: 10.1002/brb3.1864, PMID: 32989916 PMC7749543

[6] Zhang F Yu Ye Luo C Yang M Huang Z . Vestibular rehabilitation training was applied to the clinical study of patients with persistent postural-perceptual dizziness. Chin Med innovation. (2022) 19:150–3.

[7] Cao P . Comparative study on the efficacy of escitalopram and duloxetine hydrochloride in treating persistent postural dizziness. (master's thesis) Hebei North University, Zhangjiakou, China (2020).

[8] Li S Li L . Clinical effect of acupuncture combined with Lingnan fire needle puncture at Baihui point in treating chronic subjective dizziness. J Integrated Traditional Chin Western Med Cardiovasc Cerebrovascular Dis. (2021) 19:3587–90.

[9] Lin X Chen J He Y Liang H Lin Y . Effect of stimulation therapy of cerebellar apex nucleus on chronic subjective dizziness. Contemp Chin Med. (2020) 27:28–30, 35.

[10] Mo W . Effectiveness of escitalopram in treating chronic subjective dizziness and its effect on DHI score and negative emotion of patients. Northern Med. (2019) 16:90–1.

[11] Zhao H Xu L Gong X Han Q Wang X . Observation on the efficacy of sertraline hydrochloride combined with psychological intervention and visual desensitization training in the treatment of persistent postural perceptual dizziness. J Brain Neurological Diseases. (2020) 28:86–90.

[12] Zhao X Li W . Treatment of 29 cases of persistent postural-perceptual dizziness with acupoint stimulation combined with Western medicine. Hunan J Traditional Chin Med. (2021) 37:53–6.

[13] Chen D Wang H Yi R . The effect of drug therapy combined with vestibular rehabilitation and psychological intervention on HAMA HAMD DHI score in patients with chronic subjective dizziness. Shanxi Med J. (2018) 47:2658–60.

[14] Zhou R Yang L Lai X . Clinical observation on the efficacy of escitalopram oxalate combined with biofeedback-cognitive behavioral therapy in the treatment of chronic subjective dizziness. Contemp Med. (2019) 25:106–9.

[15] Qi D Yang J Chen K . Clinical efficacy of combination vestibular rehabilitation training and cognitive behavioral therapy in treating chronic subjective dizziness with anxiety. J Pract Cardiopulmonary Vasc Dis. (2018) 26:89–92.

[16] Cao H Zhai H Zhao C Gao L Zhang L . To analyze the effectiveness of rehabilitation training combined with drug treatment for persistent postural-perceptual dizziness. Contemp Med Theory. (2024) 22:16–9.

[17] Liu Z Tian Xu Cui G Yu X . Study on the clinical effect of vestibular rehabilitation training combined with duloxetine on persistent postural-perceptual dizziness with anxiety and depression. Psychol Monthly. (2024) 19:128–30.

[18] Yuan Ye Wang M Lin Y . Clinical study on the treatment of persistent postural-perceptual dizziness with acetylmorphine tablets combined with Deanxit. Neuropathy Funct reconstruction. (2020) 15:481–2.

[19] Xu J Wei C Wang Y Yang J Wei J Zhang Q . Clinical efficacy analysis of vestibular rehabilitation training in the treatment of moderate to severe persistent postural-perceptual dizziness. Chin J Clin Neurosci. (2023) 31:43–8.

[20] Liu X Wang S Hong Li . The effect of betahistine combined with vestibular rehabilitation training on balance function in patients with moderate to severe persistent postural-perceptual dizziness. Rare Dis J. (2024) 31:108–109 + 121.

[21] Hou H . Effect analysis of the combined application of eszopiclone and vestibular rehabilitation training in the treatment of chronic subjective dizziness. Chin Health Care. (2021) 39:167–9.

[22] Meng L . The clinical value of specific vestibular rehabilitation program in treating persistent postural perception dizziness. World Compendium Med. (2021) 7:10–13 + 22.

[23] Teh CSL Abdullah NA Kamaruddin NR Mohd Judi KB Fadzilah I Prepageran N . MEND therapy: A home-based option for persistent postural-perceptual dizziness. B-ENT. (2024) 20:34–44. doi: 10.5152/B-ENT.2024.231331 35794811

[24] Yang M Duan Z Zhang X Fu C . Clinical observation on the efficacy of citalopram combined with vestibular rehabilitation training and cognitive behavioral therapy in treating chronic subjective dizziness. Western Med. (2018) 30:888–91.

[25] Im JJ Na S Kang S Jeong H Lee ES Lee TK . A randomized, double-blind, sham-controlled trial of transcranial direct current stimulation for the treatment of persistent postural-perceptual dizziness (PPPD). Front Neurol. (2022) 13:868976. doi: 10.3389/fneur.2022.868976, PMID: 35493817 PMC9046552

[26] Edelman S Mahoney AEJ Cremer PD . Cognitive behavior therapy for chronic subjective dizziness: a randomized, controlled trial. Am J Otolaryngology. (2012) 33:395–401. doi: 10.1016/j.amjoto.2011.10.009, PMID: 22104568

[27] Li H Li S Lu J Zhong Hu . Clinical observation of acupuncture needle combined with rehabilitation technology in the treatment of persistent postural-perceptual dizziness. Chin folk Ther. (2022) 30:28–31.

[28] David EA Shahnaz N . Posturographic sensory ratios provide evidence for neuroplasticity after computerized vestibular rehabilitation therapy in a single group interventional trial. J Neuroeng Rehabil. (2025) 22:81. doi: 10.1186/s12984-025-01608-w, PMID: 40217271 PMC11987360

[29] Jacobson GP Newman CW . The development of the Dizziness Handicap Inventory (DHI) and its use in the evaluation of patients with dizziness and other balance disorders. J Am Acad Audiol. (1991) 2:77–87. 1837740

[30] Karas SM Barrow R Conant C Freedman JM Jacobsen PL Jemison J . MM120 (lysergide) in a controlled clinical setting: treatment of generalized anxiety disorder without co-occurring psychotherapeutic intervention. Eur Psychiatry. (2025) 68:S92–3. doi: 10.1192/j.eurpsy.2025.289

[31] Longo UG Papalia R De Salvatore S Marinozzi A Piergentili I De Lalli A . Establishing the minimum clinically significant difference (MCID) and the patient acceptable symptom score (PASS) for the hospital anxiety and depression scale (HADS) in patients with rotator cuff disease and shoulder prosthesis. J Clin Med. (2023) 12:1540. doi: 10.3390/jcm12041540, PMID: 36836074 PMC9967741

[32] Bredesen DE Amos EC Canick J Ackerley M Raji C Fiala M . Reversal of cognitive decline in Alzheimer’s disease. Aging (Albany NY). (2016) 8:1250–8. doi: 10.18632/aging.100981, PMID: 27294343 PMC4931830

[33] Zang J Zheng M Chu H Yang X . Additional cognitive behavior therapy for persistent postural-perceptual dizziness: a meta-analysis. Braz J Otorhinolaryngology. (2024) 90:101393. doi: 10.1016/j.bjorl.2024.101393, PMID: 38350404 PMC10867767

[34] Suica Z Behrendt F Ziller C Gäumann S Schädler S Hilfiker R . Comparative effectiveness of non-pharmacological treatments in patients with persistent postural-perceptual dizziness: a systematic review and effect sizes analyses. Front Neurol. (2024) 15:1426566. doi: 10.3389/fneur.2024.1426566, PMID: 39070052 PMC11272556

[35] Piatti D De Angelis S Paolocci G Minnetti A Manzari L Verdecchia DH . The role of vestibular physical therapy in managing persistent postural-perceptual dizziness: A systematic review and meta-analysis. J Clin Med. (2025) 14:5524. doi: 10.3390/jcm14155524, PMID: 40807145 PMC12347945

