# Supplementary figures and images for: Effect of conservative therapy for persistent postural-perceptual dizziness: a systematic review and meta-analysis
Source: Front Psychiatry. 2025 Oct 30;16:1676218. doi: 10.3389/fpsyt.2025.1676218 (PMC12612630; doi:10.3389/fpsyt.2025.1676218)

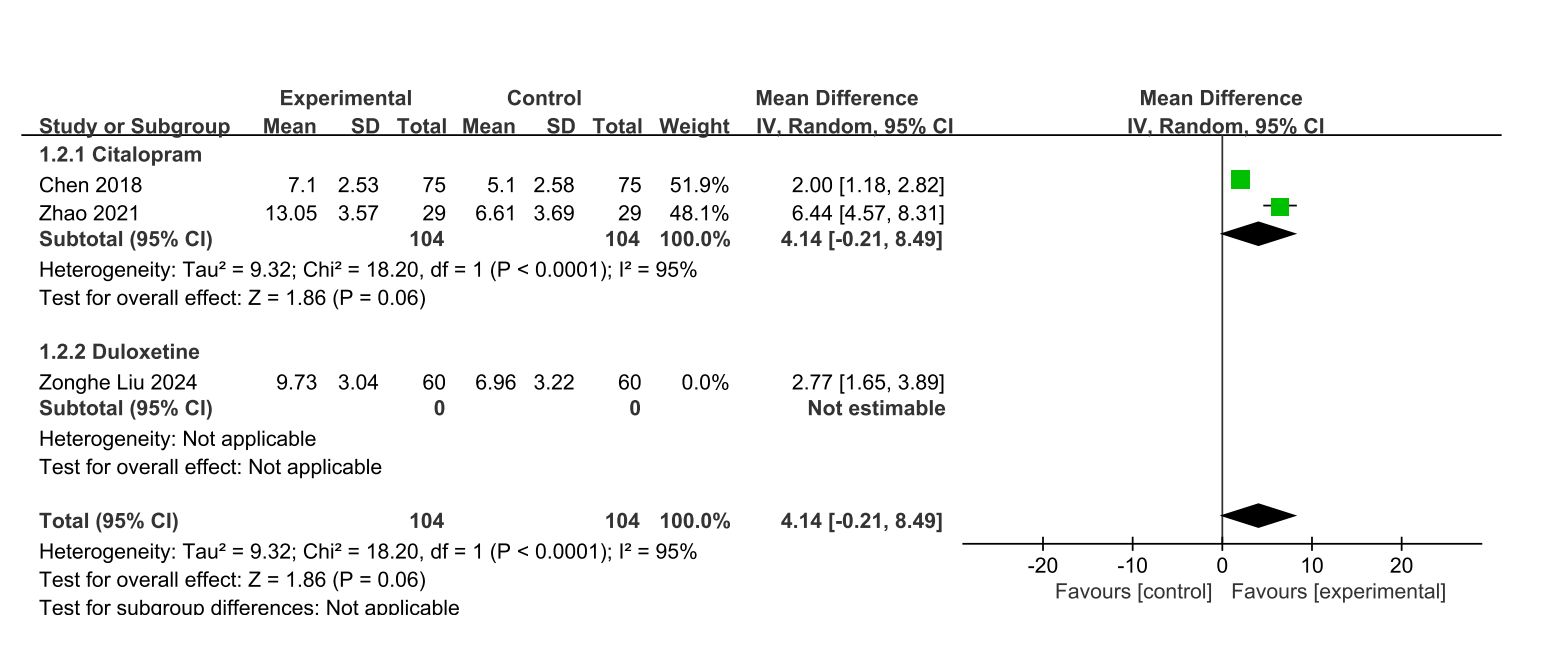

Supplement: Supplementary file 1 [file Image1.jpg]
