# Supplementary material for: Effect of conservative therapy for persistent postural-perceptual dizziness: a systematic review and meta-analysis
Source: Front Psychiatry. 2025 Oct 30;16:1676218. doi: 10.3389/fpsyt.2025.1676218 (PMC12612630; doi:10.3389/fpsyt.2025.1676218)
Supplement: Supplementary file 3 [file SupplementaryFile2.docx]

**Search Strategy in Chinese database**

**1.CNKI**

(SU=‘持续性姿势-知觉性头晕 + 持续性姿势-感知性头晕 + 慢性前庭综合征’ or TI=‘持续性姿势-知觉性头晕 + 持续性姿势-感知性头晕 + 慢性前庭综合征’ or KY=‘持续性姿势-知觉性头晕 + 持续性姿势-感知性头晕 + 慢性前庭综合征’ or AB=‘持续性姿势-知觉性头晕 + 持续性姿势-感知性头晕 + 慢性前庭综合征’) AND (SU='随机' or TI='随机' or KY='随机' or AB='随机' )

**2.WangFang**

(题名或关键词: (("持续性姿势-知觉性头晕" or "持续性姿势-感知性头晕" or "慢性前庭综合征" ) and (随机)))

**3.CBM**

((("持续性姿势-知觉性头晕"[常用字段] OR "持续性姿势-感知性头晕"[常用字段] ) OR "慢性前庭综合征"[常用字段] )) AND ("保守治疗"[常用字段] OR "非手术疗法"[常用字段]) AND ( "随机"[常用字段] OR 临床试验[文献类型] OR 随机对照试验[文献类型])

**Search Strategy in English database**

1. **PubMed**

#1 "PPPD"[Title/Abstract] OR "Persistent Postural-Perceptual Dizziness"[Title/Abstract] OR "Functional dizziness"[Title/Abstract] OR "Chronic subjective dizziness"[Title/Abstract]

#2 "randomized controlled trial"[Title/Abstract] OR "trial"[Title/Abstract] OR "randomly"[Title/Abstract] OR "placebo"[Title/Abstract] OR "randomization"[Title/Abstract] OR "randomized controlled trial"[Publication Type] OR "controlled clinical trial"[Publication Type]

#3 "non-surgical treatment"[Title/Abstract] OR "conservative treatment"[Title/Abstract]

#4 #1 OR #2 OR #3

1. **Embase**

#1 'conservative treatment'/exp

#2 'Persistent Postural-Perceptual Dizziness'/exp

#3 'randomized controlled trial'/exp

#4 'non-surgical treatment':ab,ti OR 'conservative treatment':ab,ti

#5 'PPPD':ab,ti OR 'Functional dizziness':ab,ti OR 'Functional dizziness':ab,ti OR 'Chronic subjective dizziness':ab,ti

#6 'randomized controlled trial':ab,ti OR 'controlled clinical trial':ab,ti OR 'clinical trials as topic':ab,ti OR 'single-blind method':ab,ti OR 'cross-over studies':ab,ti OR randomization:ab,ti OR placebo:ab,ti OR randomly:ab,ti OR trial:ab,ti

#7 #1 OR #4

#8 #2 OR #5

#9 #3 OR #6

#10 #7 AND #8 AND #9
