# Supplementary material for: Effect of conservative therapy for persistent postural-perceptual dizziness: a systematic review and meta-analysis
Source: Front Psychiatry. 2025 Oct 30;16:1676218. doi: 10.3389/fpsyt.2025.1676218 (PMC12612630; doi:10.3389/fpsyt.2025.1676218)
Supplement: Supplementary file 4 [file SupplementaryFile3.docx]

**Supplementary Document 2 - RoB 2**

Domain 1: Risk of bias arising from the randomization process

| **Domain 1. Randomization process** | | | | | |
| --- | --- | --- | --- | --- | --- |
| Study ID | 1.1 | 1.2 | 1.3 | 1.0  Algorithm result | 1.0 Assessor's Judgement |
| Hou 2021 | Y | Y | N | Low | Low |
| Meng 2021 | PY | NI | N | Some concerns | Some concerns |
| Zhang 2022 | Y | NI | N | Some concerns | Some concerns |
| Cao 2020 | Y | Y | N | Low | Some concerns |
| Li 2021 | Y | Y | N | Low | Some concerns |
| Li 2022 | Y | Y | N | Low | Low |
| lin 2020 | Y | Y | N | Low | Low |
| Mo 2019 | PY | NI | NI | Some concerns | Some concerns |
| Yuan 2020 | PY | NI | NI | Some concerns | Some concerns |
| Zhao 2020 | N | NI | Y | High | High |
| Zhao 2021 | PY | NI | PN | Some concerns | Some concerns |
| Chen 2018 | Y | Y | PN | Low | Low |
| Zhou 2019 | PY | NI | N | Some concerns | Some concerns |
| Xu 2023 | Y | Y | N | Low | Low |
| Edelman 2012 | Y | NI | N | Some concerns | Some concerns |
| Im 2022 | Y | Y | N | Low | Low |
| Teh 2024 | Y | Y | N | Low | Low |
| Qi 2018 | Y | Y | PN | Low | Low |
| Yang 2018 | Y | NI | PN | Some concerns | Some concerns |
| Cao 2024 | PY | Y | N | Low | Low |
| Liu 2024 | PY | NI | PN | Some concerns | Some concerns |
| Liu 2024 | PY | NI | PN | Some concerns | Some concerns |

Domain 2: Risk of bias due to deviations from the intended interventions

| **Domain 2. Deviations from intended interventions** | | | | | | | | | |
| --- | --- | --- | --- | --- | --- | --- | --- | --- | --- |
| Study ID | 2.1 | 2.2 | 2.3 | 2.4 | 2.5 | 2.6 | 2.7 | 2.0 Algorithm result | 2.0 Assessor's Judgement |
| Hou 2021 | PY | PY | PN | NA | NA | Y | NA | Low | Low |
| Meng 2021 | PY | N | PN | NA | NA | PY | NA | Low | Low |
| Zhang 2022 | PY | PN | PN | NA | NA | Y | NA | Low | Low |
| Cao 2020 | N | N | NA | NA | NA | Y | NA | Low | Low |
| Li 2021 | Y | PY | N | NA | NA | Y | NA | Low | Low |
| Li 2022 | Y | Y | PN | NA | NA | PY | NA | Low | Low |
| lin 2020 | Y | Y | PN | NA | NA | PY | NA | Low | Low |
| Mo 2019 | Y | Y | PN | NA | NA | PY | NA | Low | Low |
| Yuan 2020 | PN | PN | NA | NA | NA | PY | NA | Low | Low |
| Zhao 2020 | Y | Y | N | NA | NA | PY | NA | Low | Low |
| Zhao 2021 | Y | Y | PN | NA | NA | PY | NA | Low | Low |
| Chen 2018 | Y | Y | PN | NA | NA | PY | NA | Low | Low |
| Zhou 2019 | Y | Y | PN | NA | NA | PY | NA | Low | Low |
| Xu 2023 | Y | Y | PN | NA | NA | PY | NA | Low | Low |
| Edelman 2012 | PN | Y | PN | NA | NA | Y | NA | Low | Low |
| Im 2022 | N | PY | PN | NA | NA | Y | NA | Low | Low |
| Teh 2024 | PN | PN | NA | NA | NA | Y | NA | Low | Low |
| Qi 2018 | PY | Y | PN | NA | NA | Y | NA | Low | Low |
| Yang 2018 | Y | Y | PN | NA | NA | PY | NA | Low | Low |
| Cao 2024 | PY | PY | N | NA | NA | PY | NA | Low | Low |
| Liu 2024 | Y | Y | PN | NA | NA | Y | NA | Low | Low |
| Liu 2024 | PY | PY | PN | NA | NA | PY | NA | Low | Low |

Domain 3: Risk of bias due to missing outcome data

| **Domain 3. Mising outcome data** | | | | | |
| --- | --- | --- | --- | --- | --- |
| Study ID | 3.1 | 3.2 | 3.3 | 3.4 | 3.0  Assessor's judgement |
| Hou 2021 | PY | NA | NA | NA | Low |
| Meng 2021 | PY | NA | NA | NA | Low |
| Zhang 2022 | Y | NA | NA | NA | Low |
| Cao 2020 | Y | NA | NA | NA | Low |
| Li 2021 | NI | PY | NA | NA | Low |
| Li 2022 | NI | PN | PN | NA | Low |
| lin 2020 | NI | PY | NA | NA | Low |
| Mo 2019 | PY | NA | NA | NA | Low |
| Yuan 2020 | N | PN | Y | Y | High |
| Zhao 2020 | NI | PY | NA | NA | Low |
| Zhao 2021 | NI | PY | NA | NA | Low |
| Chen 2018 | NI | PY | NA | NA | Low |
| Zhou 2019 | NI | PY | NA | NA | Low |
| Xu 2023 | NI | PN | PN | NA | Low |
| Edelman 2012 | Y | NA | NA | NA | Low |
| Im 2022 | Y | NA | NA | NA | Low |
| Teh 2024 | Y | NA | NA | NA | Low |
| Qi 2018 | NI | PY | NA | NA | Low |
| Yang 2018 | NI | PY | NA | NA | Low |
| Cao 2024 | PY | NA | NA | NA | Low |
| Liu 2024 | PY | NA | NA | NA | Low |
| Liu 2024 | PY | NA | NA | NA | Low |

Domain 4: Risk of bias in measurement of the outcome

| **Domain 4. Measurement of the outcome** | | | | | | | |
| --- | --- | --- | --- | --- | --- | --- | --- |
| Study ID | 4.1 | 4.2 | 4.3 | 4.4 | 4.5 | 4.0  Algorithm result | 4.0  Assessor's Judgement |
| Hou 2021 | N | N | PN | NA | NA | Low | Low |
| Meng 2021 | N | N | PN | NA | NA | Low | Low |
| Zhang 2022 | PN | N | PY | PN | NA | Low | Low |
| Cao 2020 | N | N | PN | NA | NA | Low | Low |
| Li 2021 | N | N | PN | NA | NA | Low | Low |
| Li 2022 | N | N | PY | PN | NA | Low | Low |
| lin 2020 | PN | N | PN | NA | NA | Low | Low |
| Mo 2019 | N | N | PN | NA | NA | Low | Low |
| Yuan 2020 | N | N | PN | NA | NA | Low | Low |
| Zhao 2020 | N | N | PN | NA | NA | Low | Low |
| Zhao 2021 | N | N | PN | NA | NA | Low | Low |
| Chen 2018 | N | N | PN | NA | NA | Low | Low |
| Zhou 2019 | N | N | PN | NA | NA | Low | Low |
| Xu 2023 | PN | N | PY | PN | NA | Low | Low |
| Edelman 2012 | N | N | PN | NA | NA | Low | Low |
| Im 2022 | N | N | PN | NA | NA | Low | Low |
| Teh 2024 | N | N | N | NA | NA | Low | Low |
| Qi 2018 | N | N | PY | PN | NA | Low | Low |
| Yang 2018 | N | N | PN | NA | NA | Low | Low |
| Cao 2024 | N | N | PN | NA | NA | Low | Low |
| Liu 2024 | PN | PN | PY | N | NA | Low | Low |
| Liu 2024 | PN | N | PN | NA | NA | Low | Low |

Domain 5: Risk of bias in selection of the reported result

| **Domain 5. Selection of the reported result** | | | | | | |
| --- | --- | --- | --- | --- | --- | --- |
| Study ID | 5.1 | 5.2 | 5.3 | 5.0 Algorithm result | 5.0 Assessor's Judgement | 5.1 Assessor's Judgement |
| Hou 2021 | NI | PN | PN | Some concerns | Some concerns | Some concerns |
| Meng 2021 | NI | NI | NI | Some concerns | Some concerns | Some concerns |
| Zhang 2022 | NI | PN | PN | Some concerns | Some concerns | Some concerns |
| Cao 2020 | NI | PN | PN | Some concerns | Some concerns | Some concerns |
| Li 2021 | NI | PN | PN | Some concerns | Some concerns | Some concerns |
| Li 2022 | NI | PN | PN | Some concerns | Some concerns | Some concerns |
| lin 2020 | NI | PN | PN | Some concerns | Some concerns | Some concerns |
| Mo 2019 | NI | PN | PN | Some concerns | Some concerns | Some concerns |
| Yuan 2020 | NI | PN | PN | Some concerns | Some concerns | Some concerns |
| Zhao 2020 | NI | PN | PN | Some concerns | Some concerns | Some concerns |
| Zhao 2021 | NI | PN | PN | Some concerns | Some concerns | Some concerns |
| Chen 2018 | NI | N | N | Some concerns | Some concerns | Some concerns |
| Zhou 2019 | NI | PN | PN | Some concerns | Some concerns | Some concerns |
| Xu 2023 | NI | PN | PN | Some concerns | Some concerns | Some concerns |
| Edelman 2012 | Y | PN | PN | Low | Low | Low |
| Im 2022 | PY | PN | PN | Low | Low | Low |
| Teh 2024 | Y | PN | PN | Low | Low | Low |
| Qi 2018 | NI | PN | PN | Some concerns | Some concerns | Some concerns |
| Yang 2018 | NI | PN | PN | Some concerns | Some concerns | Some concerns |
| Cao 2024 | NI | NI | NI | Some concerns | Some concerns | Some concerns |
| Liu 2024 | NI | NI | NI | Some concerns | Some concerns | Some concerns |
| Liu 2024 | NI | NI | NI | Some concerns | Some concerns | Some concerns |

Domain 6: Overall risk of bias

| **Domain 6. Overall Bias** | | |
| --- | --- | --- |
| Study ID | Algorithm's overall Judgement | Assessor's overall Judgement |
| Hou 2021 | Some concerns | Some concerns |
| Meng 2021 | Some concerns | Some concerns |
| Zhang 2022 | Some concerns | Some concerns |
| Cao 2020 | Some concerns | Some concerns |
| Li 2021 | Some concerns | Some concerns |
| Li 2022 | Some concerns | Some concerns |
| lin 2020 | Some concerns | Some concerns |
| Mo 2019 | Some concerns | Some concerns |
| Yuan 2020 | High | High |
| Zhao 2020 | High | High |
| Zhao 2021 | Some concerns | Some concerns |
| Chen 2018 | Some concerns | Some concerns |
| Zhou 2019 | Some concerns | Some concerns |
| Xu 2023 | Some concerns | Some concerns |
| Edelman 2012 | Some concerns | Some concerns |
| Im 2022 | Low | Low |
| Teh 2024 | Low | Low |
| Qi 2018 | Some concerns | Some concerns |
| Yang 2018 | Some concerns | Some concerns |
| Cao 2024 | Some concerns | Some concerns |
| Liu 2024 | Some concerns | Some concerns |
| Liu 2024 | Some concerns | Some concerns |
