# Supplementary material for: Effect of conservative therapy for persistent postural-perceptual dizziness: a systematic review and meta-analysis
Source: Front Psychiatry. 2025 Oct 30;16:1676218. doi: 10.3389/fpsyt.2025.1676218 (PMC12612630; doi:10.3389/fpsyt.2025.1676218)
Supplement: Supplementary file 5 [file SupplementaryFile4.docx]

**Appendix：GRADE Summary of Findings Tables for SSRI plus other conservative therapies versus SSRI alone in Patients with PPPD**

**Question:** [SSRI plus other conservative therapies] compared to [SSRI used alone] for [postural-perceptual dizziness]

| **Certainty assessment** | | | | | | | **№ of patients** | | **Effect** | | **Certainty** | **Importance** |
| --- | --- | --- | --- | --- | --- | --- | --- | --- | --- | --- | --- | --- |
| **№ of studies** | **Study design** | **Risk of bias** | **Inconsistency** | **Indirectness** | **Imprecision** | **Other considerations** | **[SSRI plus other conservative therapies]** | **[SSRI used alone]** | **Relative (95% CI)** | **Absolute (95% CI)** |  |  |
| **DHI** | | | | | | | | | | | | |
| 8 | randomised trials | serious | serious | not serious | not serious | none | 640 | 319 | - | MD **8.42 higher** (6.18 higher to 10.66 higher) | ⨁⨁◯◯ Low |  |
| **HAMA** | | | | | | | | | | | | |
| 3 | randomised trials | serious | serious | not serious | not serious | none | 328 | 164 | - | MD **3.57 higher** (1.48 higher to 5.65 higher) | ⨁⨁◯◯ Low |  |
| **HAMD** | | | | | | | | | | | | |
| 3 | randomised trials | serious | serious | not serious | not serious | none | 328 | 164 | - | MD **3.38 higher** (2.2 higher to 4.55 higher) | ⨁⨁◯◯ Low |  |
| **HADS** | | | | | | | | | | | | |
| 3 | randomised trials | serious | serious | not serious | serious | none | 188 | 93 | - | MD **6.21 higher** (0.37 higher to 12.04 higher) | ⨁◯◯◯ Very low |  |

**CI:** confidence interval; **MD:** mean difference

**Appendix：GRADE Summary of Findings Tables for VRT plus other conservative therapies versus VRT alone in Patients with PPPD**

**Question:** [VRT plus other conservative therapies] compared to [VRT alone] for [postural-perceptual dizziness]

| **Certainty assessment** | | | | | | | **№ of patients** | | **Effect** | | **Certainty** | **Importance** |
| --- | --- | --- | --- | --- | --- | --- | --- | --- | --- | --- | --- | --- |
| **№ of studies** | **Study design** | **Risk of bias** | **Inconsistency** | **Indirectness** | **Imprecision** | **Other considerations** | **[VRT plus other conservative therapies]** | **[VRT alone]** | **Relative (95% CI)** | **Absolute (95% CI)** |  |  |
| **DHI** | | | | | | | | | | | | |
| 2 | randomised trials | serious | not serious | not serious | not serious | none | 182 | 91 | - | MD **11.37 higher** (8.27 higher to 14.47 higher) | ⨁⨁⨁◯ Moderate |  |
| **HAMA** | | | | | | | | | | | | |
| 3 | randomised trials | serious | serious | not serious | not serious | none | 262 | 131 | - | MD **4.75 higher** (4.03 higher to 5.46 higher) | ⨁⨁◯◯ Low |  |
| **HAMD** | | | | | | | | | | | | |
| 2 | randomised trials | serious | not serious | not serious | not serious | none | 182 | 91 | - | MD **6.36 higher** (5.34 higher to 7.38 higher) | ⨁⨁⨁◯ Moderate |  |

**CI:** confidence interval; **MD:** mean difference
