# Supplementary material for: Effect of conservative therapy for persistent postural-perceptual dizziness: a systematic review and meta-analysis
Source: Front Psychiatry. 2025 Oct 30;16:1676218. doi: 10.3389/fpsyt.2025.1676218 (PMC12612630; doi:10.3389/fpsyt.2025.1676218)
Supplement: Supplementary file 6 [file SupplementaryFile5.docx]

**Sensitivity analyses of DHI for SSRI**


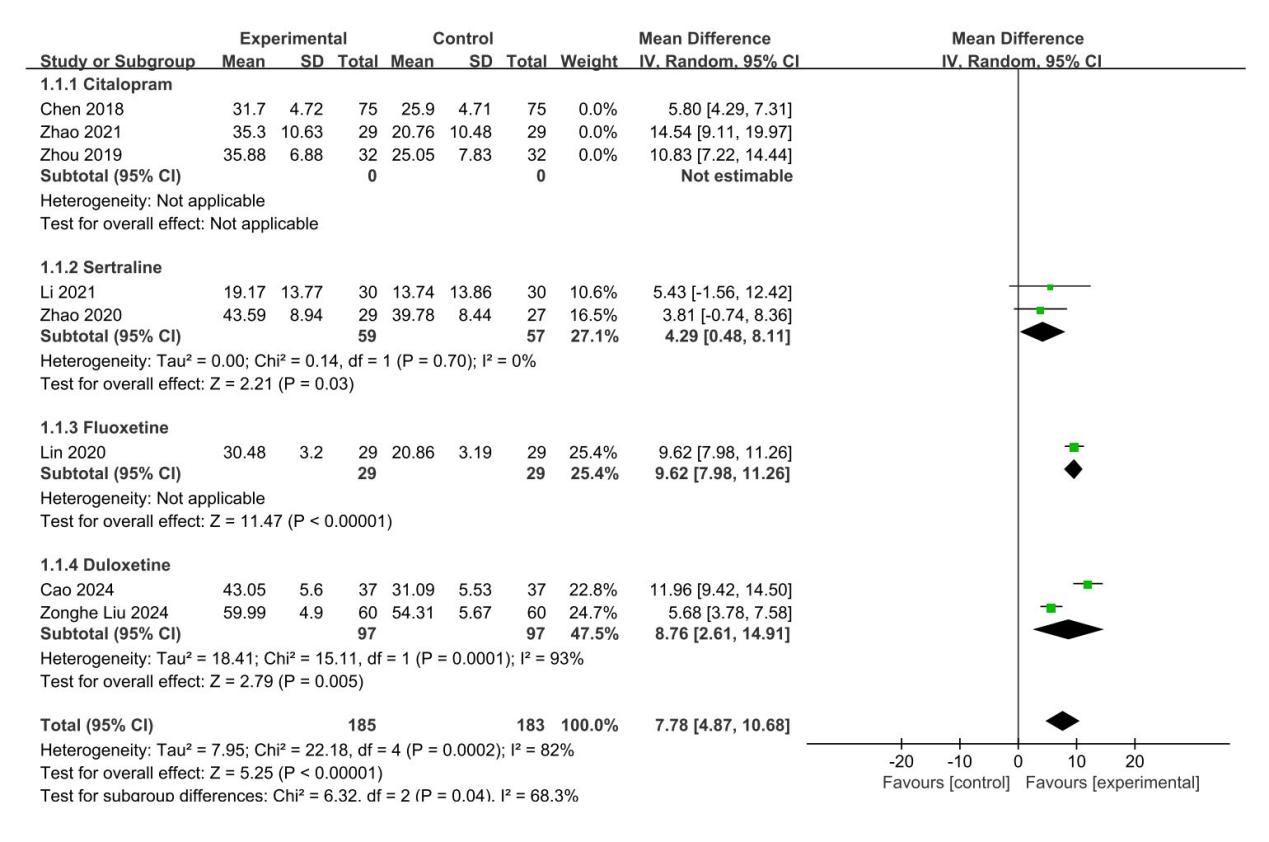


**Figure 1: Removing the result of citalopram**

**
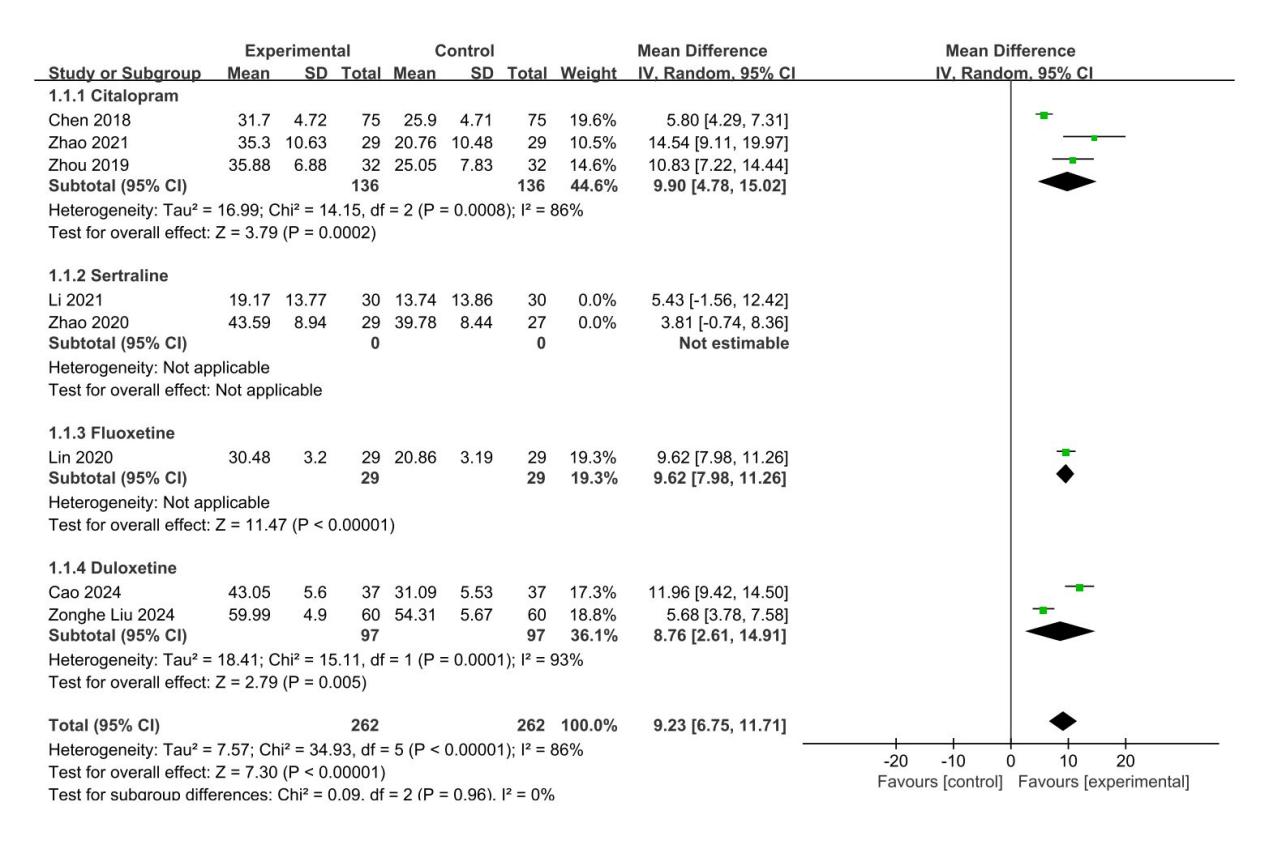
**

**Figure 2: Removing the result of sertraline**


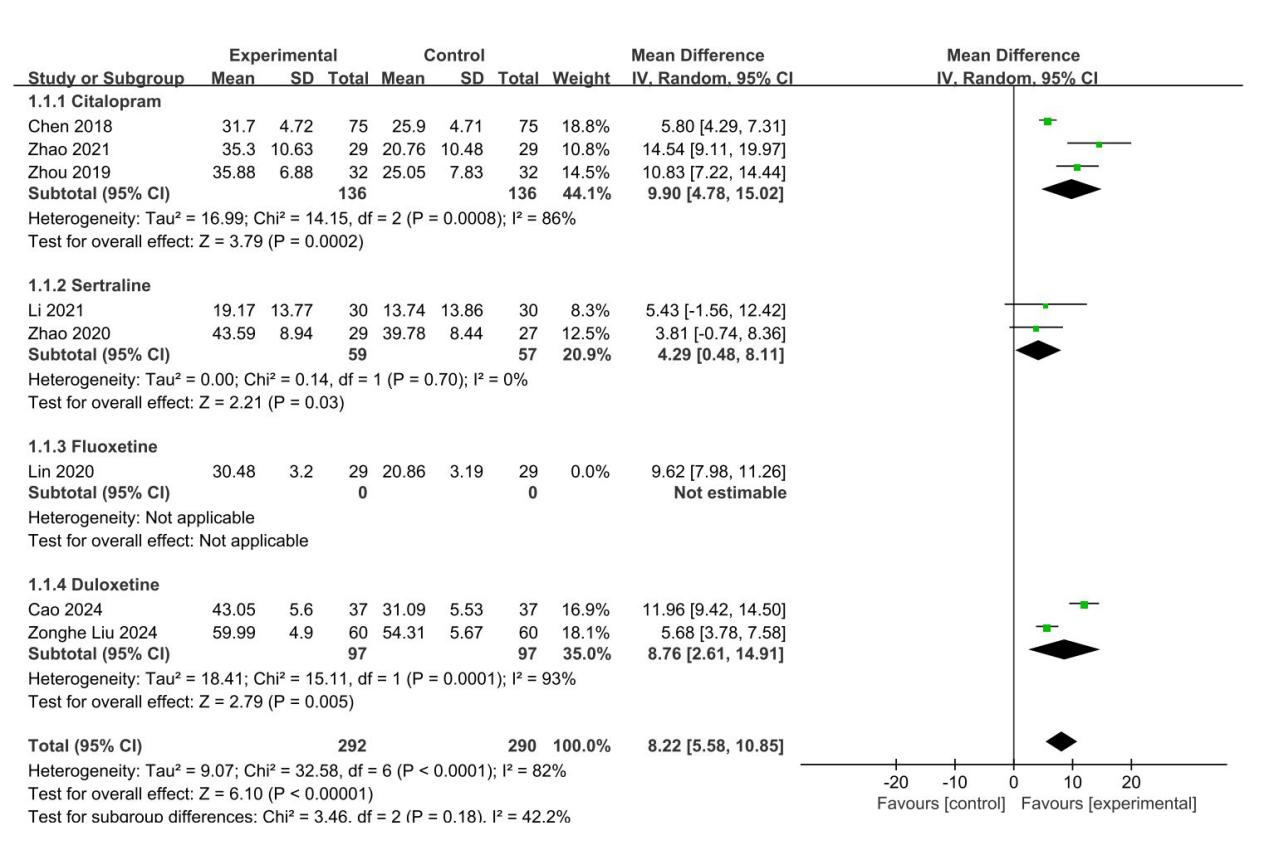


**Figure 3: Removing the result of fluoxetine**

**
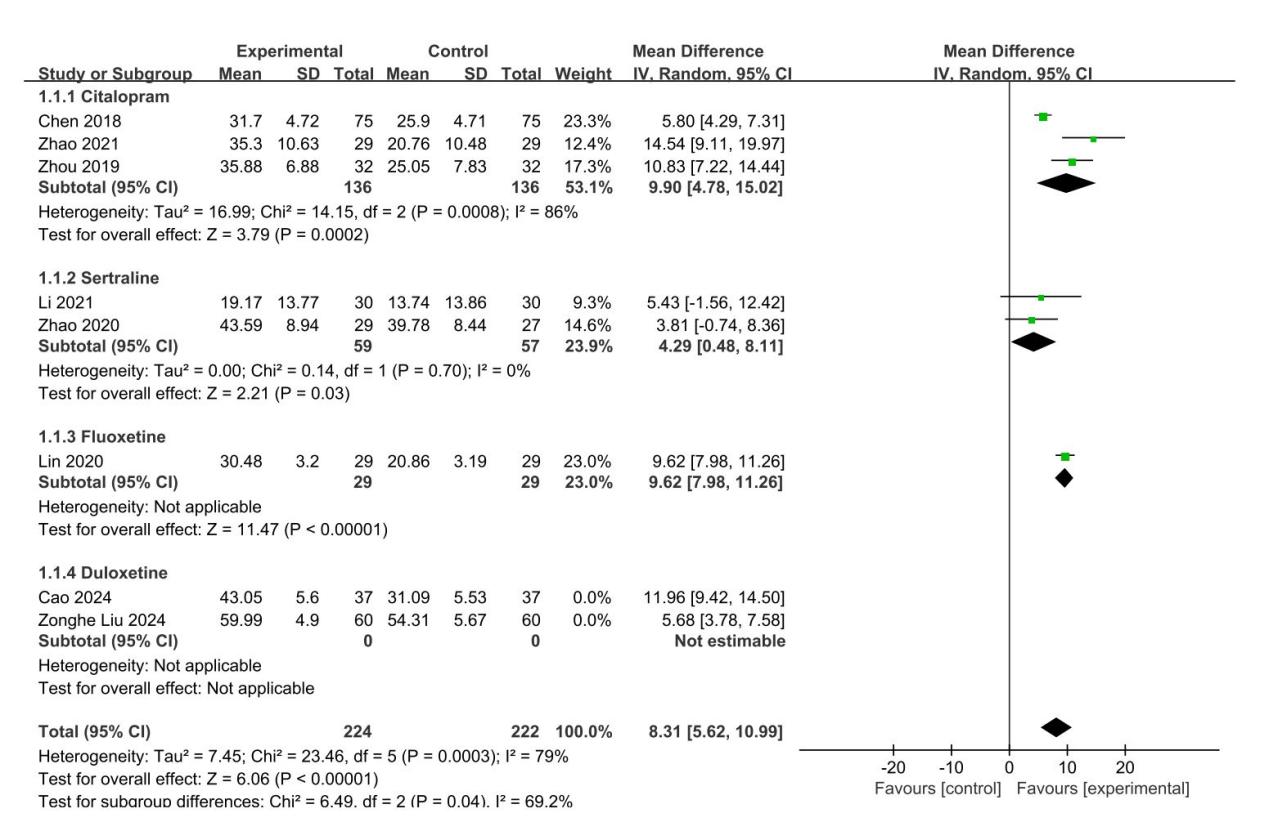
**

**Figure 4: Removing the result of duloxetine**
